# Molecular Profiling of Docetaxel-Resistant Prostate Cancer Cells Identifies Multiple Mechanisms of Therapeutic Resistance

**DOI:** 10.3390/cancers13061290

**Published:** 2021-03-14

**Authors:** Thiago S. Lima, Diego Iglesias-Gato, Luciano D. O. Souza, Jan Stenvang, Diego S. Lima, Martin A. Røder, Klaus Brasso, José M. A. Moreira

**Affiliations:** 1 Department of Drug Design and Pharmacology, Faculty of Health and Medical Sciences, University of Copenhagen, 2100 Copenhagen, Denmark; thiago_hias@hotmail.com (T.S.L.); diego.iglesias@cpr.ku.dk (D.I.-G.); luciano.souza@sund.ku.dk (L.D.O.S.); stenvang@sund.ku.dk (J.S.); 2 CAPES Foundation, Ministry of Education of Brazil, Brasília 70040-020, Brazil; 3 Sino-Danish Center for Education and Research (SDC), Aarhus University, 8000 Aarhus C, Denmark; 4 Department of Cytogenetics, Albert Sabin Children’s Hospital, Fortaleza 60410-794, Brazil; diego_silva_lima@yahoo.com.br; 5 Copenhagen Prostate Cancer Center, Department of Urology, Copenhagen University Hospital, 2100 Copenhagen, Denmark; Martin.Andreas.Roeder@regionh.dk (M.A.R.); klaus.brasso@regionh.dk (K.B.)

**Keywords:** prostate cancer, docetaxel resistance, cellular models, androgen independence

## Abstract

**Simple Summary:**

Therapeutic options for the treatment of men with metastatic castration-resistant prostate cancer are limited. Docetaxel—a taxane-based chemotherapeutic agent—was the first treatment to demonstrate significant efficacy in the treatment of this disease. However, responses to docetaxel are frequently curtailed by development of drug resistance, and patients eventually succumb to disease progression due to acquisition of drug resistance. In this study, we established drug-resistant prostate cancer cell lines and identified several mechanisms that may be associated with the development of drug resistance in prostate cancer. Actioning these mechanisms could provide a potential approach to re-sensitize drug-resistant cancer cells to docetaxel treatment and thereby further add to the life-prolonging effects of this drug in men with metastatic castration-resistant prostate cancer.

**Abstract:**

Docetaxel—a taxane-based chemotherapeutic agent—was the first treatment to demonstrate significant improvements in overall survival in men with metastatic castration-resistant prostate cancer (mCRPC). However, the response to docetaxel is generally short-lived, and relapse eventually occurs due to the development of resistance. To explore the mechanisms of acquired docetaxel resistance in prostate cancer (PCa) and set these in the context of androgen deprivation therapy, we established docetaxel-resistant PCa cell lines, derived from the androgen-dependent LNCaP cell line, and from the LNCaP lineage-derived androgen-independent C4-2B sub-line. We generated two docetaxel-resistant LNCaPR and C4-2BR sub-lines, with IC50 values 77- and 50-fold higher than those of the LNCaP and C4-2B parental cells, respectively. We performed gene expression analysis of the matched sub-lines and found several alterations that may confer docetaxel resistance. In addition to increased expression of ABCB1, an ATP-binding cassette (ABC) transporter, and a well-known gene associated with development of docetaxel resistance, we identified genes associated with androgen signaling, cell survival, and overexpression of ncRNAs. In conclusion, we identified multiple mechanisms that may be associated with the development of taxane drug resistance in PCa. Actioning these mechanisms could provide a potential approach to re-sensitization of docetaxel-resistant PCa cells to docetaxel treatment and thereby further add to the life-prolonging effects of this drug in men with mCRPC.

## 1. Introduction

Prostate cancer (PCa) is the most common cancer among men in developed countries and a major cause of male mortality, mostly due to disease progression to metastatic castration-resistant prostate cancer (mCRPC) [1,2]. Although several chemotherapeutic drugs have been tested in mCRPC, the first one to have shown significant benefit in terms of overall survival was docetaxel, a microtubule-stabilizing taxane [3,4,5,6,7]. The survival benefit demonstrated in mCRPC was short (2–3 months) but significant, and docetaxel is still frequently used as standard first-line treatment of mCRPC [8]. Unfortunately, responses are invariably limited in time and patients eventually succumb to disease progression because of acquired drug resistance; 3-year progression-free survival rates for docetaxel treatment are below 1% [9]. Although the mechanisms through which PCa becomes resistant to docetaxel are not completely understood, previous studies have identified multiple mechanisms of resistance. These include, but are not limited to, enhanced intracellular drug extrusion activity mediated by members of the family of adenosine triphosphate-binding cassette (ABC) transporters, such as ABCB1/P-glycoprotein (P-gp) [10], modulation of cell death processes such as apoptosis and autophagy [11,12], mutations in β-tubulin [13], and modified AR signaling [14,15].

To identify mechanisms of resistance that could lead to the discovery of biomarkers able to predict docetaxel resistance, or of novel therapeutic targets, we developed and characterized docetaxel-resistant PCa cell models. Given that docetaxel is primarily relevant for treatment of PCa in the metastatic setting, we used the LNCaP and C4-2B PCa cell lines, as these lines constitute a cellular model of PCa progression that mimics the natural history of the disease. LNCaP is a poorly tumorigenic, androgen-sensitive, and non-metastatic PCa cell line, derived from a needle biopsy taken from the left supraclavicular lymph node of a 50-year-old Caucasian male patient [16]. A castration-resistant sub-line, C4-2, was isolated from a tumor that developed from LNCaP cells injected into castrated nude mice [17,18], and a metastatic derivative of C4-2, termed B, was later isolated from a bone metastasis that developed after orthotopic transplantation of C4-2 cells in nude mice. Together, the LNCaP and C4-2B cell lines constitute a model of cancer progression, from localized, androgen-dependent PCa to mCRPC, which closely mimics the disease [19]. We generated two docetaxel-resistant LNCaP and C4-2B sub-lines (LNCaP^R^ and C4-2B^R^, respectively) and characterized them in terms of proliferation, viability, gene dosage, cell cycle, and resistance to docetaxel. We also performed genome-wide gene expression profiling of the two parental PCa cell lines (LNCaP and C4-2B) and their docetaxel-resistant derivatives and identified a number of mechanisms that may be associated with the development of taxane drug resistance.

## 2. Materials and Methods

### 2.1. Cell Culture and Resistant Cell Lines Development

LNCaP and C4-2B PCa cells, kindly provided by Prof. Flores-Morales (Department of Drug Design and Pharmacology, Faculty of Health and Medical Sciences, University of Copenhagen, 2100 Copenhagen, Denmark), were cultured and maintained in RPMI-1640 medium + glutaMAXTM-I (Gibco, Invitrogen, Thermo Scientific, Hvidovre, Denmark) supplemented with 10% fetal bovine serum (FBS). LNCaP and C4-2B docetaxel-resistant cell lines were generated by exposing them to an initial dose of 2.5 nM docetaxel (suspended in dimethyl sulfoxide (DMSO)) for 48–72 h. After each treatment, cells were allowed to fully recover before assessing their resistance to docetaxel and any experimental work. LNCaP cells were exposed to docetaxel through 38 passages over 320 days at concentrations ranging from 2.5–70 nM. C4-2B cells were exposed to docetaxel through 45 passages over 315 days at concentrations ranging from 2.5–100 nM. Untreated parental cells were cultured alongside as an appropriate control to ensure the resistant phenotype alterations. Batches of cells in different passages and statuses of docetaxel resistance were frozen down after assessing their resistance. All cell lines were tested for mycoplasma contamination using Mycoplasma PCR detection kit (GATC-Biotech, Köln, Germany) and no infections were observed.

### 2.2. Gene Expression Analysis and Microarray Profiling

RNA expression was assessed using human Affymetrix Clariom D arrays (Eurofins Genomics Labor AROS, Galten, Denmark). Arrays were analyzed using the SST-RMA algorithm in the Affymetrix Expression Console Software. Differentially expressed genes were identified using significance analysis of microarrays (SAM) to control the false positive error rate [20]. All genes resulting from this analysis met a 5% false discovery rate (FDR). The list of significant transcripts was assessed by the DAVID Bioinformatics Resources (https://david.ncifcrf.gov/), a web-based statistical hypergeometric test applied for enrichment analysis of Gene Ontology (GO) categories. The datasets generated and/or analyzed during the current study are available from the corresponding author on reasonable request.

### 2.3. Cytotoxicity Assay

Cytotoxicity was performed using the 3-(4,5-dimethylthiazol-2-yl)-2,5-diphenyl-tetrazolium bromide (MTT) assay as previously described [8]. Briefly, cells were plated at a density of 8000 cells/well in triplicates in a 96-well cell culture plate and allowed to grow over 24 h prior to treatment. After 48 or 72 h of treatment, drugs were removed and 0.5 mg/mL MTT (Sigma-Aldrich, Søborg, Denmark) was added to each well. Following incubation for three hours, 20% sodium dodecyl sulphate (SDS) in 0.02 M hydrochloric acid (HCL) was added to each well in order to dissolve the formed formazan crystals overnight. Absorbance of formazan was measured in a microplate spectrophotometer (PowerWaveX, Bio-Tek Instruments, Swindon, UK) at 570 nm and the background absorbance of MTT was measured at 670 nm. Cell viability was calculated in percent compared to untreated control cells.

### 2.4. Real-Time Cell Proliferation Monitoring

Cell adherence and proliferation were monitored in real time using the xCELLigence system real-time cell analysis (RTCA) E-Plate (Agilent, Glostrup, Denmark). The xCELLigence RTCA DP instrument was used according to the manufacturer’s instructions. Briefly, after equilibration to 37 °C, E-plates were placed into the xCELLigence RTCA DP instrument, and the baseline impedance was determined to ensure that all connections were working within acceptable limits across the plate. For each experiment, 10,000 LNCaP and 8000 C4-2B cells were seeded in each well of an E-plate. The impedance value of each well was automatically monitored by the xCELLigence system for a duration of 140 h and is presented as a normalized cell index value.

### 2.5. Cell Cycle Analysis by Flow Cytometry

Cells were plated for 24 h in 6-well plates at a density of 200,000 cells/well. After plating for 24 h, serum starved in RPMI-1640 medium + glutaMAXTM-I (Gibco, Invitrogen, Carlsbad, CA, USA) was added for an additional 24 h to synchronize the cells. Cells were treated for 24 h, followed by wash with PBS and trypsinization. The single cell suspension was collected and centrifuged at 1200 rpm for 5 min at 5 °C, and pellets were resuspended and fixed with 96% cold ethanol. Samples were centrifuged and resuspended in 100 μL staining buffer containing 0.25% Triton (Sigma-Aldrich) in PBS, 25 μg/mg RNase A (Qiagen, Hilden, Germany), and 10 μg/mL PI (Sigma-Aldrich) and incubated for 30 min at 37 °C. Cell cycle progression was measured by BD FACSVerseTM flow cytometer (BD Bioscience, Albertslund, Denmark). Data were analyzed in Flowjo 10.1 Single cell Analysis Software (TreeStar, Ashland, OR, USA).

### 2.6. Protein Extraction, Western Blotting, and Nanocapillary Electrophoresis

Whole cells were harvested and lysed using lysate buffer M-PER Mammalian Protein Extraction Reagent (Thermo Scientific, Hvidovre, Denmark) supplemented with Pierce Protease and Phosphatase Inhibitor Mini tables (Thermo Scientific). Cell lysates were centrifuged at 14,000× *g* for 10 min at 4 °C and supernatants were collected. Total amount of protein was assessed by the PierceTM BCA Protein Assay Kit (Thermo Scientific), according to the manufacturer’s instructions. The Novex^®^ NuPAGE^®^ MES SDS Running Buffer (Thermo Scientific) was used for separation of proteins according to manufacturer’s instructions. Samples were loaded onto precast 10- or 15-well 4–12% Bis-Tris Gel gels (Novex^®^ NuPAGE^®^, Invitrogen). Proteins were blotted onto a nitrocellulose membrane (iBlot^®^2 NC, Invitrogen) using an iBlot^®^2 gel transfer device. Blots were blocked for 1 h in washing buffer (PBS + 0.1% Tween 20) containing 5% non-fat dry milk and incubated overnight with the appropriate primary antibody diluted in blocking reagent: Anti-PgP (Abcam, Cambridge, UK); Anti-TRAIL antibody (MAB687) (R&D systems, Lille, France); Anti-β-Actin Antibody (Sigma-Aldrich; Purified Mouse Anti-Human Caspase-3 (C3172) (BD Bioscience); anti-cleaved Caspase-3 (Cell Signaling, #9664); Purified Mouse Anti- P150 (BD Transduction Laboratories™, Albertslund, Denmark). After washing 3 × 10 min in TBS-T, membranes were incubated with horseradish peroxidase-conjugated secondary antibody (Mouse/Rabbit) for 1 h at RT. Membranes were washed 3 × 10 min in TBS-T and developed using Clarity Western ECL substrate (Bio-Rad) detection reagent. Protein bands were detected with the UVP BioSpectrum Imaging System (UVP, Thermo Scientific). The Peggy SueTM system (Protein Simple, San Jose, CA, USA) was used to detect and quantify Anti-PgP (Abcam, Cambridge, UK) and Anti- AR Antibody (441): sc-7305 (Santa Cruz Biotech, Santa Cruz, CA, USA), both diluted 1:50. Samples were prepared and loaded in a 384-well assay plate according to the manufacturer’s instructions, and data were analyzed using Compass software Compass for SW softwareVersion 5.0.1, Protein Simple, Oxford, UK).

### 2.7. Immunostaining

For the immunocytochemistry, cells were fixed in 10% PBS buffered formalin (VWR) and left to incubate at 4 °C overnight before being cut into 3 μm sections and placed on glass slides. After being deparaffinized and rehydrated, sections were exposed to induced antigen retrieval to unmask epitopes. Sections were boiled for 10 min in Envision Flex Target Retrieval Solution, high pH (Dako, Glostrup, Denmark), diluted 1:50 in miliQ H_2_O, before being incubated for 1 h with primary ABCB1 antibody (Abcam) diluted 1:1000 in antibody diluent with background reducing components (Dako). Then, sections were washed twice in TBS + 0.5% Triton X-100 and incubated for 20 min with a High Definition Polymer Detector (AH diagnostics, Tilst, Denmark). Colorimetric signals were detected using DAB. Sections were developed with EnvisionTM FLEX DAB + Chromogen (Dako) diluted in EnvisionTM FLEX Substrate Buffer to visualize the primary antibody. Sections were counterstained with Mayer’s hematoxylin (Hounisen, Skanderborg, Denmark) and mounted with Pertex xylene-based mounting media (Hounisen).

### 2.8. Cytogenetic Analysis

Parental and docetaxel-resistant cells lines (at similar passage) were harvested and the chromosomes were banded as described previously [21]. Nomenclature follows ISCN recommendations. In agreement with ISCN recommendations, chromosome abnormalities were classified as clonal if two or more metaphases has an identical structural abnormality, or three or more metaphases had gained or lost a specific chromosome. Metaphases were analyzed using CytoVision software (applied spectral imaging; CytoVision v. 7.0, Leica-Microsystems, São Paulo, Brazil).

### 2.9. Fluorescence In Situ Hybridization

*ABCB1* gene copy was assessed by FISH using LNCaP and C4-2B, both parental and resistant cell lines. Cells and slides were prepared as previously described [22]. FISH was performed, using probes developed by Empire Genomics (Williamsville, NY, USA) specific for the region containing the *ABCB1* gene and centromere of chromosome 7, according to the manufacturer’s instructions. A fluorescence microscope (Zeiss AX10; Carl Zeiss A/S, Birkerød, Denmark) with a Texas Red/FITC double filter was used for quantifying red (*ABCB1*) and green (chromosome 7) signals.

## 3. Results

### 3.1. Establishment of Docetaxel-Resistant PCa Cell Lines

**Figure 1:**
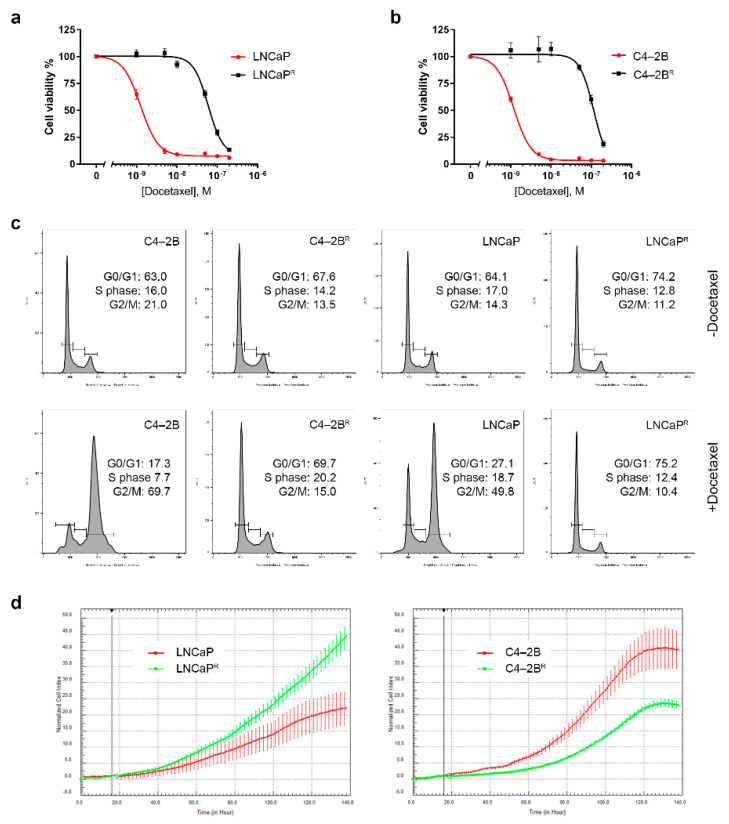
Docetaxel-resistant LNCaP^R^ and C4-2B^R^ cells. (**a**,**b**) Determination of IC_50_ values of LNCaP^R^ and C42B^R^ cells, respectively. Cell viability was measured using a standard MTT assay, following exposure of cells to varying concentrations of docetaxel for 72 h. All plotted values are normalized to the untreated control. (**c**) Cell cycle profiles of LNCaP^R^ and C4-2B^R^ cells treated with docetaxel. After treatment with vehicle (DMSO) or 20 nM of docetaxel for 24 h, cells were harvested, fixed, and stained with PI before being analyzed by FACS. Data were analyzed using FlowJo software (BD Biosciences. (**d**) Real-time monitoring of cell growth rates with xCELLigence of LNCaP/LNCaP^R^ and C4-2B/C4-2B^R^.

The LNCaP cell line and its derivative, C4-2B, constitute a preclinical model of PCa progression, from the poorly tumorigenic, androgen-sensitive, and non-metastatic LNCaP cell line to the highly aggressive, androgen- insensitive, and metastatic C4-2B cell line [17]. We generated sub-lines with acquired drug resistance to docetaxel (C4-2B^R^ and LNCaP^R^, respectively) by continuous exposure (over a period of 10 months) of C4-2B and LNCaP cells to sub-lethal, stepwise increasing concentrations of docetaxel (from 0.1 to 100 nM). Cell viability assays established docetaxel IC_50_ values of 99.47–100.50 and 49.50–50.65 nmol/L for C4-2B^R^ and LNCaP^R^, respectively, compared to the C4-2B and LNCaP parental cells that had IC_50_ values of 1.00–1.40 and 0.78–1.06 nmol/L, respectively (Figure 1a,b, respectively). In other words, docetaxel IC_50_ values in C4-2B^R^ and LNCaP^R^ cells were increased by 77- and 50-fold, respectively, compared to their corresponding parental cell lines.

True resistance to docetaxel of C4-2B^R^ and LNCaP^R^ cells was confirmed by characterizing their cell cycle profiles upon docetaxel exposure. We performed flow cytometry analysis of propidium iodide (PI)-stained cells exposed, or not, to 20 nM docetaxel. Exposure to docetaxel for 24 h induced G2/M arrest in both the C4-2B and LNCaP parental cells, while no significant effect was observed on the resistant cells (Figure 1c), demonstrating that the C4-2B^R^ and LNCaP^R^ cells not only survived exposure to docetaxel but were also able to proliferate in its presence. Cell proliferation rates were monitored using a label-free, real-time cell analysis platform (xCELLigence). As shown in Figure 1d, in the docetaxel-resistant LNCaP^R^ line, growth rates were slightly higher than those of the parental cell line (Figure 1d, left-hand panel, compare green line with red line), whereas the docetaxel-resistant C4-2B^R^ cells (Figure 1d, right-hand panel, green line) had a lower proliferation rate than the parental drug-sensitive cell line (Figure 1d, right-hand panel, red line).

### 3.2. Identification of Differentially Expressed Genes in Docetaxel-Resistant Cell Lines

To identify those genes that may contribute to docetaxel resistance in C4-2B^R^ and LNCaP^R^ cells, we performed transcriptome analysis using RNA microarrays (Clariom D arrays; ~28,000 features) of three independent passages of C4-2B^R^ and LNCaP^R^ and the matched parental C4-2B and LNCaP cells, respectively. Cells were grown in the absence of docetaxel to identify stably acquired changes.

We found a total of 1300 significantly deregulated genes in C4-2B^R^ cells compared to the matched parental C4-2B cell line, whereas only 152 genes were significantly deregulated in LNCaP^R^ compared to the matched parental LNCaP cell line. A complete list of deregulated genes in both cell lines is provided as supplementary data (Tables S1 and S2). A total of 30 genes showed a common pattern of deregulation between the two cell lines (Table 1 and Table 2).

**Figure 2:**
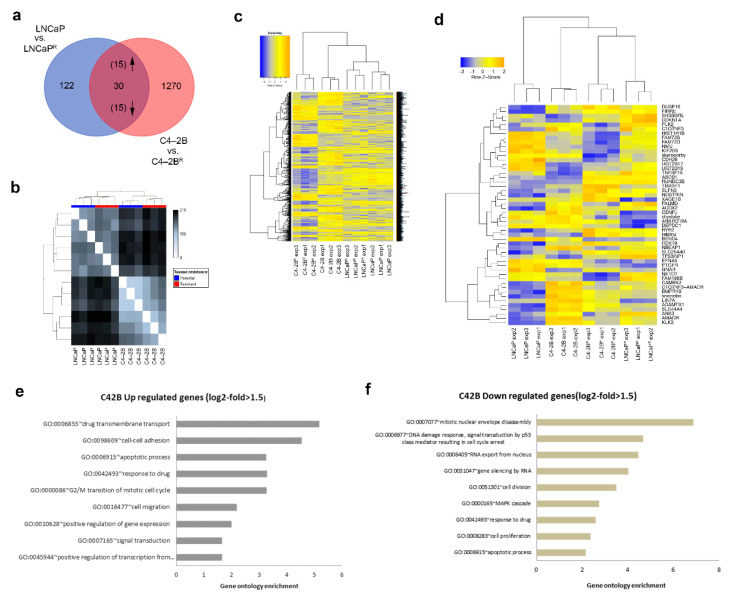
Gene expression analysis of docetaxel-resistant cells. (**a**) Venn diagram showing the number of differentially expressed genes in the docetaxel-resistant lines compared to the parental lines. Only genes with at least 1.5-fold change in expression levels between the parental and docetaxel-resistant cells (*p* ≤ 0.05) were selected. (**b**) Distance matrix graph of analyzed samples. Distance between two samples was calculated using Euclidean metrics. (**c**) Heatmap of the 1300 genes that are significantly differentially expressed between parental and C4-2B^R^ docetaxel-resistant cells and the 152 genes differentially expressed between parental and LNCaP^R^ resistant cells. (**d**) Heatmap including only the genes that are differentially expressed by both LNCaP^R^ and C4-2B^R^ resistant cells. Heatmap visualization was performed using the gplots package in R. The intensity of the squares reflects the fold repression (blue) or fold induction (orange), according to the color scale at the top. (**e**,**f**) Selected Gene Ontology (GO) categories for the C4-2BR cell line genes, for (**e**) up- and (**f**) down-regulated genes.

Among the 30 commonly deregulated genes, 15 were consistently up-regulated and 15 consistently down-regulated when comparing resistant and parental cells (Figure 2a, Table 1 and Table 2). Relationships between samples were determined by pairwise comparison and a distance matrix calculated using principal component analysis (PCA) (Figure 2b), showing that the cell of origin was the most important parameter driving gene profiles. Explorative unsupervised clustering analysis revealed a good segregation of the arrays in their respective classes on the basis of expression values, either based on cell lines: C4-2B vs. LNCaP, or based on phenotype: sensitive (parental) vs. resistant (Figure 2c,d). To identify possible biological pathways involved in docetaxel resistance, Gene Ontology (GO) enrichment analysis of the genes differentially expressed in C4-2B^R^ resistant cells compared to the matched parental C4-2B cell line was performed (Figure 2e,f).

We identified several biological processes known to be involved in docetaxel resistance, such as drug transmembrane transport and apoptosis, among others. Up- or down-regulation of these processes is of particular interest as it reflects stable, inheritable changes to gene expression, rather than transient alterations in direct response to drug exposure. Due to the limited number of gene expression changes, Gene Ontology analysis of LNCaP^R^ and parental cells was not informative, as only few biological processes were significantly enriched, and these were driven by a small set of genes.

### 3.3. Differential Androgen Receptor Signaling Behavior in Docetaxel-Resistant C4-2B^R^ and LNCaP^R^ Cell Lines

**Figure 3:**
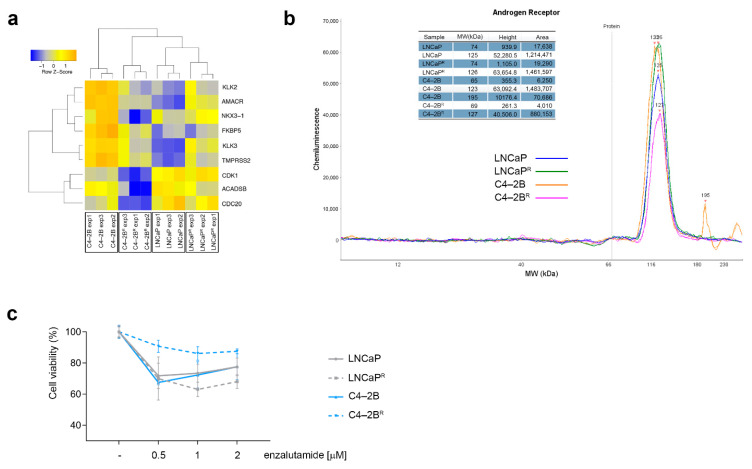
Androgen receptor signaling profiling of docetaxel-resistant cell lines. (**a**) Heatmap illustrating AR canonical target genes differentially expressed in the two docetaxel-resistant cell lines. (**b**) Androgen receptor protein levels assessed by nanocapillary electrophoresis (**c**) Effect of enzalutamide on LNCaP^R^ and C4-2B^R^ resistant cells. LNCaP and C4-2B, and their docetaxel-resistant matched derivatives LNCaP^R^ and C4-2B^R^, were treated with varying concentrations of enzalutamide for 72 h and cell viability was measured using MTT assay and normalized to an untreated control.

Since alterations in androgen receptor (AR) signaling are known to be critical in PCa progression, and resistance to docetaxel has been associated with deregulated AR signaling [14,15], we specifically examined the expression pattern of nine canonical AR target genes (*KLK3*, *TMPRSS2*, *NK3X-1*, *KLK2*, *AMACR*, *CDC2O*, *CDK1*, *FKBP5*, and *ACADSB*) in our docetaxel-resistant sub-lines. We found cell line-specific changes in the profile of the AR-regulated genes, concomitant with acquisition of docetaxel resistance. In the androgen-independent C4-2B^R^ line, AR-regulated genes were significantly down-regulated compared to the matched parental cell line (Figure 3a). By contrast, no significant changes in transcriptional activity of canonical AR-regulated genes were found in the androgen-sensitive and docetaxel-resistant LNCaP^R^ cells (Figure 3a). Further, expression of *AR* mRNA itself was decreased in the androgen-independent C4-2B^R^ cells compared to the matched parental cell line (1.97-fold; *p* < 0.01), whereas we found no differential expression of AR in docetaxel-resistant LNCaP^R^ cells compared to the matched parental cell line (1.04-fold; *p* = 0.84).

Given the discordance between *AR* mRNA and AR protein levels previously observed in LNCaP cells [23], and since AR protein degradation is a key regulatory mechanism in prostate epithelial cells [24], we examined the levels of AR protein expression in our docetaxel-resistant cell lines. To ensure precise and accurate quantification of AR protein expression levels, we used an automated capillary immunoassay system that allows superior accuracy as compared to conventional Western blot analysis [25]. We found that AR protein expression was decreased in the C4-2B^R^ cells (1.7-fold) while it increased slightly in the LNCaP^R^ cells (1.2-fold) compared to their respective matched parental cell lines (Figure 3b). Of note, both docetaxel-resistant cell lines displayed detectable AR protein expression, suggesting that AR signaling is maintained, albeit at different levels, following acquisition of docetaxel resistance. To determine whether the differential expression of AR and AR-canonical target genes in the docetaxel-resistant cells translated into different responses to treatment with anti-androgens, both parental and resistant cells were grown in the presence of various concentrations of the non-steroidal antiandrogen enzalutamide, and cell viability was assessed after 72 h. As expected, the docetaxel-resistant C4-2B^R^ cells, which down-regulated AR signaling, showed a significantly lower sensitivity to enzalutamide as compared to the C4-2B parental cells (Figure 3c; *p* < 0.0001, two-way ANOVA). The LNCaP^R^ docetaxel-resistant cells, on the other hand, which up-regulated AR signaling, did not differ significantly from the parental cells or, if anything, were only slightly more sensitive to enzalutamide than the matched parental cells (Figure 3c).

### 3.4. Increased ABCB1 Expression Is a Common Feature of the Docetaxel-Resistant Sub-Lines

**Figure 4:**
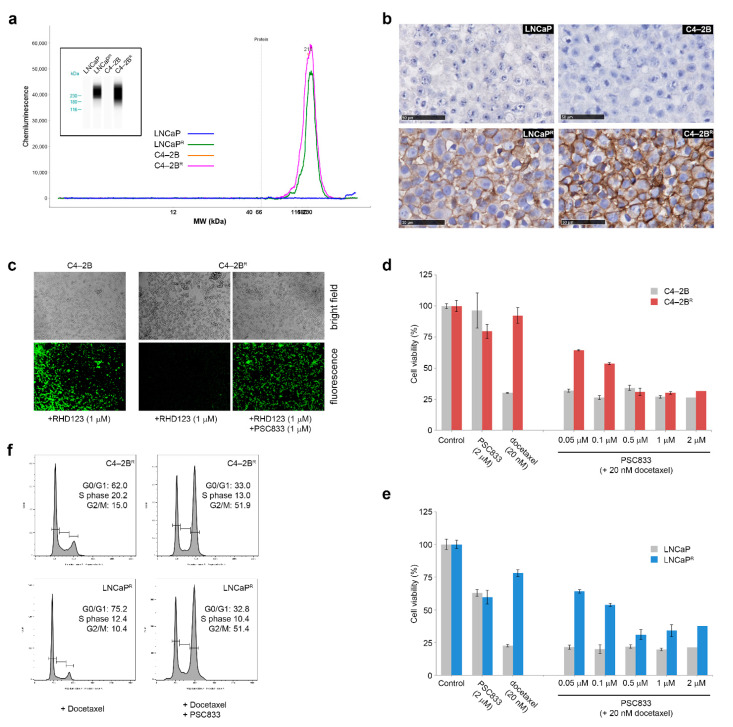
ABCB1 expression levels in the docetaxel-resistant cell lines. (**a**) ABCB1 expression on total protein cellular extracts was assessed by nanocapillary electrophoresis using β-actin expression as normalization factor. (**b**) ABCB1 expression was also investigated by immunocytochemistry, showing expression in docetaxel-resistant cells and a strong membrane presence. (**c**) Representative images of Rhodamine 123 accumulation in C4-2B^R^ cells, untreated or after blockage of ABCB1 function with PSC833. Magnification, 10×. (**d**,**e**) Blocking ABCB1 with PSC833 reverts resistance to docetaxel. Cells were treated for 48 h with vehicle, PSC833 alone, docetaxel alone, or a combination of 20 nM docetaxel and increasing doses of PSC833, showing that the resistance phenotype was dependent on ABCB1 function. (**f**) Flow cytometric analyses of cell cycle progression of C4-2B^R^ and LNCaP^R^ resistant cells treated with 20 nM of docetaxel alone or in combination with 1 μM PSC833 for 24 h. Cells were harvested, fixed, and stained with PI for fluorescence-activated cell sorter analysis. Data were analyzed using FlowJo Software.

To address common and, presumably, more prevalent mechanisms of resistance, we examined the 30 genes we found to be deregulated in both cell lines (Table 1 and Table 2). *ABCB1*, also called multiple drug resistance 1 (*MDR1*) gene, was found to be up-regulated at the mRNA level in both the C4-2B^R^ and LNCaP^R^ docetaxel-resistant cell lines. ABCB1/MDR1 is an ATP-dependent efflux pump that decreases the intracellular concentration of a variety of anti-cancer drugs, leading to multidrug resistance in several types of cancer, including PCa [10,26]. Western blot analysis using automated capillary immunoassay measurements demonstrated a robust overexpression of ABCB1 in both the C4-2B^R^ and LNCaP^R^ docetaxel-resistant cell lines (Figure 4a). Overexpression of ABCB1 was further established by immunohistochemistry, which confirmed up-regulation and showed that ABCB1 located predominantly at the cell membrane in these cell lines (Figure 4b). We then examined the functional activity of ABCB1 in the resistant cells as an efflux pump, by measuring the efflux of an ABCB1-specific substrate—the fluorescent tracer dye rhodamine-123 (RHD123)—in these cells [27]. Parental and matched resistant cells were loaded with RHD123 (1 μM) for 1 h, and intracellular levels of RHD123 were assessed by fluorescence microscopy after 30 min of efflux time (illustrated for C4-2B and C4-2B^R^ in Figure 4c). Due to the low levels of ABCB1 present in the parental C4-2B cells, RHD123 accrued in the uptake phase was almost completely retained by these cells after 30 min of efflux time (Figure 4c, C4-2B). Resistant cells, unlike the parental cells, held almost no intracellular RHD123 after just 30 min of efflux (Figure 4c, C4-2B^R^ +RHD123). To establish that these differences in RHD123 accumulation were due to efflux activity by ABCB1, resistant cells were exposed to PSC833, a cyclosporin A analog and ABCB1 inhibitor [28], prior to efflux. Exposure to PSC833 caused resistant cells to retain intracellular RHD123, showing that overexpression of ABCB1 in resistant cells was associated with increased efflux activity (Figure 4c, C4-2B^R^ +RHD123 +PSC833).

To further establish the contribution of ABCB1 overexpression on docetaxel resistance in C4-2B^R^ and LNCaP^R^ cells, we measured cell viability after docetaxel treatment for 48 h in parental and resistant cells while concomitantly blocking ABCB1 with increasing concentrations of PSC833 (Figure 4d,e). Combining docetaxel and PSC833 fully re-sensitized C4-2B^R^ and partially decreased cell viability in LNCaP^R^ cells to docetaxel when combined with 0.5 μM of PSC833. As our resistant cell lines were able to bypass docetaxel-induced G2/M cell cycle arrest, we also investigated if treatment with docetaxel combined with PSC833 could affect cell cycle progression. As shown in Figure 4f, ABCB1 inhibition combined with docetaxel treatment could induce G2/M cell cycle arrest on resistant cells, demonstrating that ABCB1 was a major contributor, although not the only one, to docetaxel resistance in our model systems.

**Figure 5:**
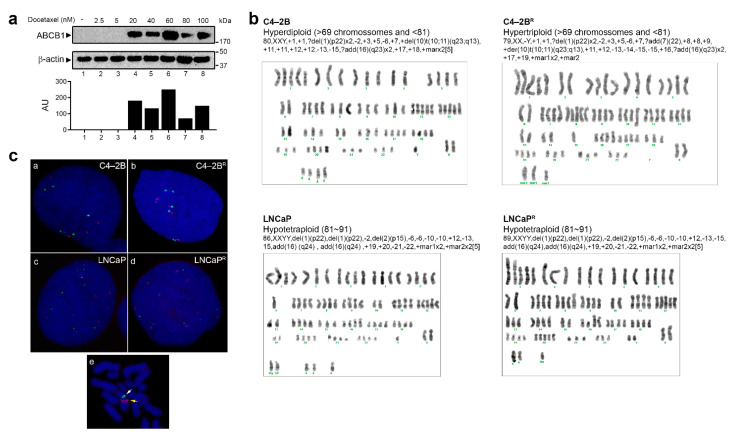
Karyotypic and gene level evaluation of ABCB1 copy number in drug-resistant cell lines. (**a**) Western blot analysis of ABCB1 expression in consecutive passages of LNCaP and C4-2B cells generated during the process of acquisition of drug resistance. Full Western blot image can be found in Figure S1. Lower panel: graphs depicting densitometry measurements of ABCB1 levels relative to those of β-actin in arbitrary units (AU). (**b**) Representative G-band karyotypic analyses of the parental and docetaxel-resistant cell lines. (**c**) Fluorescence in situ hybridization (FISH) using probes specific for chromosome 7 centromere (Green) and *ABCB1* gene (Red) was performed in C4-2B (subpanel a), C4-2B^R^ (subpanel b), LNCaP (subpanel c), and LNCaP^R^ (subpanel d) parental and resistant cell lines, respectively. Subpanel e shows a representative image of metaphase FISH of an LNCaP cell, confirming that the probes used recognized discrete regions in the same chromosome.

Although reduced efficacy of taxanes has been previously associated with ABCB1 expression, the specific mechanisms responsible for increased expression of ABCB1 in acquired drug resistance are not completely clear. Different mechanisms of *ABCB1* gene induction, such as alterations in gene dosage or transcriptional regulation, have been proposed. The human *ABCB1* gene is located on the chromosomal 7q21.1 region [29]. Abnormalities in this region, caused by genomic instability and chromosomal rearrangements, may result in genomic amplification and increased copy number of the *ABCB1* gene, which may result in increased expression of *ABCB1* [22,30,31,32]. During the process of establishment of the resistant phenotype, we stored sequential batches of resistant cells with intermediate resistance to docetaxel. To further clarify the mechanism behind the constitutive overexpression of *ABCB1* in our model system, we examined different sequential batches of resistant C4-2B^R^ cells. As shown in Figure 5a, expression of *ABCB1* was abruptly increased concomitant with an increase in the maximum tolerable dose of docetaxel from 5 to 20 nM. From 20 to 100 nM (highest tolerable concentration), no major differences were seen on *ABCB1* expression, indicating that the final levels of resistance to docetaxel were a result of a compounded effect of multiple mechanisms, one of which was *ABCB1* up-regulation. It was conceivable that this abrupt increase in *ABCB1* expression was due to chromosome 7 abnormalities [33]. To determine if alterations in gene dosage could be responsible for overexpression of *ABCB1* in our model system, karyotypical analyses of metaphase chromosomes, stained by Giemsa for G-bands, were performed on parental as well as resistant cell lines. However, as shown in Figure 5b, no significant differences were found with regard to either numeric or structural chromosome 7 alterations that would support an increased copy number of the *ABCB1* gene and therefore overexpression of *ABCB1*. Both C4-2B^R^ and LNCaP^R^ cells maintained the parental chromosome 7 ploidy, despite an up-regulation of *ABCB1* mRNA expression of 20-fold and 10-fold, respectively.

Since G-band karyotyping only allows detection of aberrations that involve gains or losses of portions of the genome and rearrangements >3 Mb of DNA [34], fluorescence in situ hybridization (FISH) was performed to further confirm the same DNA content in parental and resistant cells. Using probes specific for chromosome 7 centromere and *ABCB1*, no gene copy number gain difference was observed (Figure 5c), suggesting that another mechanism than gene dosage, for instance, transcriptional up-regulation, post-transcriptional regulation, or epigenetic modifications, was responsible for up-regulation of *ABCB1* in our resistant cell lines.

### 3.5. Modulation of Cell Death Mechanisms May Contribute to Multifactorial Docetaxel Resistance

Intensified drug efflux due to *ABCB1* overexpression was a contributing factor to the docetaxel resistance phenotype of C4-2B^R^ and LNCaP^R^ cells, but not the only one. This was particularly evident in LNCaP^R^ cells, where other mechanisms of docetaxel resistance must have contributed for cells to have reached higher levels of resistance. One such mechanism may relate to the ability of resistant cells to evade drug-induced cell death due to activation of anti-apoptotic mechanisms [35]; co-deregulation of ABCB1 and apoptotic proteins was previously shown to correlate with a multifactorial resistant phenotype in cancer cells [11,36].

Our gene expression analysis identified two non-protein-coding RNA (ncRNA) paralogues, vtRNA1-1 and vtRNA1-2, as being strongly up-regulated in the C4-2B^R^ docetaxel-resistant cells as compared to their parental counterpart, with log2 ratios of 139 and 20, respectively. In a recent study, Amort and colleagues found that vtRNA1-1 is able to inhibit both the extrinsic and intrinsic apoptotic pathways [37] and convincingly showed that the anti-apoptotic effect of vtRNA1-1 is an intrinsic feature of this ncRNA and independent of the vault complex. Furthermore, vtRNA1-1 has been shown to regulate the autophagic flux by direct interaction with the autophagy receptor protein p62/sequestosome-1 [38]. Thus, overexpression of vtRNAs may constitute a mechanism of drug resistance by opposing cell death in the C4-2^R^ docetaxel-resistant cells.

**Figure 6:**
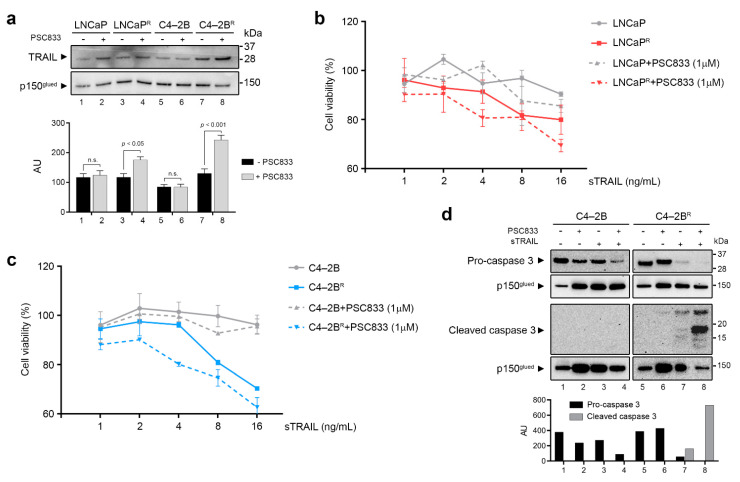
TRAIL status and its role in docetaxel-resistant prostate cancer (PCa) cell lines (**a**) Parental and resistant cells were cultured in the presence or absence of the ABCB1 inhibitor PSC833 for 48 h and total cellular protein extracts were used to assess endogenous TRAIL expression by Western blot. Full Western blot image can be found in Figure S2. Upper panel: representative immunoblot showing levels of TRAIL protein present in whole cell extracts. P150 was used as normalizing control. Lower panel: graphs depicting pooled densitometry measurements of TRAIL levels relative to those of p150 in arbitrary units (AU). Data points are presented as mean ± SEM of triplicate measurements. (**b**) Cell viability was measured using MTT assay and normalized to an untreated control. LNCaP and C4-2B were treated with increasing doses of sTRAIL for 48 h. (**c**) LNCaP^R^ and C4-2B^R^ were treated for 48 h with vehicle, PSC833 alone, sTRAIL alone, or a combination of 1 μM PSC833 and increasing doses of sTRAIL. (**d**) Procaspase-3 and activated caspase-3 expression was assessed by Western blot after a pretreatment with PSC833 alone, sTRAIL alone, and PSC833 combined with sTRAIL. Lower panel: graphs depicting densitometry measurements of Procaspase-3 and activated caspase-3 levels relative to those of p150 in arbitrary units (AU). Full Western blot image can be found in Figure S3.

We further scrutinized our data for genes related to cell death pathways and found that the Tumor Necrosis Factor superfamily member 10 (*TNFSF10/TRAIL*) gene was significantly up-regulated in LNCaP^R^ cells compared to its parental counterpart (Table S2). Expression of *TNFSF10/TRAIL* was also increased in the C4-2B^R^ docetaxel-resistant cells (1.72-fold), albeit to a much lower extent. However, Western blot analysis revealed TRAIL protein overexpression not only in LNCaP^R^ cells but also in C4-2B^R^ cells compared to their matched parental counterparts (Figure 6a).

We then examined whether expression of TRAIL was affected by ABCB1 efflux function in our docetaxel-resistant cell lines. Blocking ABCB1 activity with PSC833 (1 μM) for 48 h induced TRAIL protein expression further in both docetaxel-resistant cell lines (Figure 6a), which is consistent with previous reports that showed endogenous TRAIL expression levels to be associated with ABCB1 efflux activity [39]. Although this association between TRAIL and ABCB1 was previously reported, it was uncertain whether ABCB1 up-regulation confers resistance or sensitivity to TRAIL-targeted therapies [39,40].

TRAIL activates the extrinsic apoptosis pathway by binding to its functional death receptors and induces apoptotic cell death in cancer cells. TRAIL, or agonist antibodies to TRAIL receptors, can cause apoptosis of cancer cells with little or no toxicity, and, consequently, TRAIL is an attractive therapeutic possibility. We assessed whether our docetaxel-resistant cell lines, which overexpressed ABCB1, were sensitive to TRAIL-induced cytotoxicity. After treatment of resistant and parental cells with increasing concentrations of soluble recombinant human TRAIL (sTRAIL), we found that C4-2B^R^ cells (Figure 6b, left-hand panel, red stippled line) were much more sensitive to sTRAIL than the parental cells (Figure 6b, left-hand panel, gray solid line). LNCaP^R^ cells (Figure 6b, right-hand panel, blue stippled line), on the other hand, did not demonstrate any significant difference on cell viability compared to the matched parental cell line (Figure 6b, right-hand panel, gray solid line).

To further evaluate whether ABCB1 efflux activity, rather than expression, was a determinant of sTRAIL sensitivity, we measured the cytotoxic effect of sTRAIL on parental and resistant cells while blocking ABCB1 efflux activity (Figure 6c), using activation of caspase-3 as an additional readout (Figure 6d). We found that blocking ABCB1 efflux did not change the effect of sTRAIL on C4-2B^R^ or LNCaP^R^ cells.

## 4. Discussion

We generated a set of docetaxel-resistant PCa cell lines (LNCaPR and C4-2BR) based on a cellular model of PCa progression (Figure 1). The resulting cell lines, C4-2B^R^ and LNCaP^R^, together with their respective parental lines, C4-2B and LNCaP, respectively, constitute useful cellular models to study docetaxel resistance in PCa in the context of disease progression from localized, hormone-dependent PCa to mCRPC. Gene expression profiles of these resistant models identified likely mechanisms involved in acquisition of resistance to docetaxel (Figure 2 and Supplementary Tables S1 and S2). We discovered at least three different mechanisms contributing to the resistance phenotype that may be of clinical relevance: modulation of AR signaling (Figure 3), up-regulation of the ABCB1 drug efflux pump (Figure 4 and Figure 5), and deregulation of genes associated with cell death mechanisms (Supplementary Tables S1 and S2 and Figure 6).

### 4.1. Modulation of AR Signaling

Our data showing that resistance to docetaxel was associated with AR signaling are consistent with results from the CHAARTED (ChemoHormonal Therapy versus Androgen Ablation Randomized Trial for Extensive Disease in Prostate Cancer) and STAMPEDE (Systemic Therapy in Advancing or Metastatic Prostate Cancer: Evaluation of Drug Efficacy) clinical trials, which suggested an association between androgen receptor signaling and docetaxel sensitivity in PCa [6,41]. Secondary data analysis of the CALGB 90401 study (a trial evaluating addition of bevacizumab to docetaxel and prednisone in mCRPC) [42] demonstrated that a greater decline in androgens during docetaxel therapy was associated with a greater overall survival [43], indicating that clinical improvement may be a result of docetaxel-mediated androgen suppression during treatment, and suggesting that resistance to docetaxel may affect androgen suppression.

We found distinct differences in the expression patterns of AR and AR-regulated genes in connection with acquisition of docetaxel resistance, in C4-2B^R^ cells, compared to LNCaP^R^ cells. Given that the LNCaP cell line represents the androgen-sensitive and non-metastatic setting of PCa, whereas C4-2B embodies the metastatic and androgen-independent setting of the disease, inference from our model would suggest that development of resistance to docetaxel may lead to enhanced or suppressed AR signaling, depending on the setting of the disease, which has implications for clinical decision making. As novel treatments become available, physicians need to decide how best to sequence available treatments. Currently, there are no conclusive clinical data providing direct evidence on how best to sequence post-docetaxel treatments in CRPC. If our model system holds true, patients with CRPC who received docetaxel, when developing resistance, will become less responsive to hormonal treatments, such as enzalutamide. Indeed, enzalutamide showed a better effect in the post-docetaxel setting in our model, when docetaxel was used in the androgen-sensitive setting (Figure 3), indicating that the association between docetaxel treatment and AR signaling needs to be further studied.

### 4.2. Up-Regulation of the ABCB1 Drug Efflux Pump

We also found that overexpression of the ABCB1 efflux pump was strongly associated with development of docetaxel resistance in both cell lines in our model system (Figure 4). Overexpression of ABC transporters, such as ABCB1, which efflux chemotherapeutic drugs, such as docetaxel, out of cancer cells is a widespread mechanism of resistance that plays a significant role in treatment failure, including in PCa [10,26]. Cytogenetic alterations and increased copy number of the *ABCB1* gene have been linked to increased expression of *ABCB1* and acquisition of drug resistance [22,30,31,32,33]. However, we found no evidence of cytogenetic alterations, chromosomal 7q21.1 region amplification, or *ABCB1* gene duplications in our drug-resistant cell lines (Figure 5), indicating another mechanism was responsible for up-regulation of *ABCB1*. Suggestively, the *RUNDC3B* gene was one of the most up-regulated genes in both cell lines—by 107-fold in C4-2B^R^ and 174-fold in LNCaP^R^. The *RUNDC3B* gene encodes a poorly characterized protein that interacts with Rap2-binding protein 9 (RPIP9), a RAS family protein involved in the MAPK cascade pathway [44]. The *RUNDC3B* gene is nested in the same genetic locus as *ABCB1*, with several *RUNDC3B* exons located in the complementary strand of the *ABCB1* gene, and expression of *RUNDC3B* is associated with alternative regulation of the ABCB1 promoter and expression of *ABCB1* mRNA isoforms [45,46,47]. All in all, these data suggest the occurrence of an event that caused a localized increase in the transcriptional activity of both *ABCB1* and *RUNDC3B* genes, such as an epigenetic event. As both cell lines displayed a similar behavior, with concomitant overexpression of *ABCB1* and *RUNDC3B* genes, it is conceivable that this mechanism may be a common one, and if it is actionable, it may provide an attractive target for preventing development of drug resistance.

### 4.3. Modulation of Anti-Apoptotic Genes

Our gene expression analysis also identified two non-protein-coding RNA (ncRNA) paralogues, vtRNA1-1 and vtRNA1-2, as being strongly up-regulated in the C4-2B^R^ docetaxel-resistant cells as compared to their parental counterparts, with log2 ratios of approximately 139 and 20, respectively. Vault RNAs can recognize and bind chemotherapeutic compounds, thus preventing them from accessing their target sites. As a result, vtRNAs have been associated with chemoresistance in cancer cells [37,48,49,50]. The vtRNAs are integral components of the vault complex, a massive 13MDa ribonucleoprotein (RNP) complex, suggested to play roles in multidrug resistance of cancer cells, and apoptosis resistance, among others [48,49,51,52]. In a recent study, Amort and colleagues found that vtRNA1-1 is able to inhibit both the extrinsic and intrinsic apoptotic pathways [37] and convincingly showed that the anti-apoptotic effect of vtRNA1-1 is an intrinsic feature of this ncRNA and independent of the vault complex. Interestingly, ectopic overexpression of vtRNA1-1 led to overexpression of TNF/TNFR superfamily members including *TNFSF10/TRAIL*, which we found up-regulated in our drug-resistant cell lines (Figure 6). Although up-regulation of TRAIL in connection with acquisition of drug resistance is counter-intuitive, it may simply be derived from the integration of multiple mechanisms affecting TRAIL expression, such as vtRNA1-1 up-regulation, AR signaling—as TRAIL is an AR target gene—and ABCB1 up-regulation, all of which affect TRAIL expression [37,39,53]. We also found a functional association between TRAIL expression and ABCB1 efflux activity (Figure 6a). Taken together, our results indicate that the expression of ABCB1 was important for cellular responses to sTRAIL, and that inhibition of ABCB1 efflux activity boosted the effectiveness of sTRAIL-mediated cell death, forming a case for a closer examination of combination therapies of TRAIL and ABCB1 inhibitors in docetaxel-resistant PCa.

We found a total of 1300 deregulated genes in C4-2B^R^ cells and 152 deregulated genes in LNCaP^R^ cells compared to the corresponding parental lines. Here, we focused primarily on a few of those genes that showed a common pattern of deregulation between the two cell lines (Table 1 and Table 2), and for which we could rationalize a cellular effect in the context of drug resistance, based on their known cellular functions. However, there were multiple additional deregulated genes (and by inference, mechanisms) that were not as straightforward to allocate to a specific effect. Take the case of cyclin-dependent kinase inhibitor 1 (*CDKN1A*), or p21^Cip1^, a cell cycle regulator and tumor suppressor, overexpressed in both LNCaP^R^ and C4-2B^R^. Increased expression of *CDKN1A* should induce cell cycle arrest and reduce proliferative activity. However, we found that LNCaP^R^ cells actually showed slightly higher proliferation rates than the parental cell line, whereas C4-2B^R^ cells had a lower proliferation rate than the corresponding parental cell line (Figure 1d), suggesting another mechanism rather than cell cycle control might be at play. In fact, overexpression of p21 has been previously shown to desensitize LNCaP cells to docetaxel treatment, identifying the p38/p53/p21 signaling axis as an important determinant of susceptibility towards docetaxel-induced apoptosis in prostate cancer [54].

## 5. Conclusions

We generated and characterized two docetaxel-resistant prostate cancer sub-lines. We, like many others previously, identified ABCB1 gene up-regulation as a common mechanism involved in acquired docetaxel resistance. However, we could also identify multiple additional mechanisms, associated with AR signaling and regulation of cell death processes, which may contribute to docetaxel resistance and could be necessary to complement up-regulation of ABCB1 to achieve resistance to high concentrations of docetaxel.

Comparison of our data with similar datasets generated from other docetaxel-resistant derivatives of C4-2B and LNCaP cells generated by other researchers, as well as from other PCa cell lines, should provide further knowledge as to whether acquisition of resistance is achieved by deregulation of a finite number of specific genes, or if one, rather, should enumerate all the traits that have been found to be associated with resistance under an overarching organizing principle, or hallmarks of resistance.

## Figures and Tables

**Table 1:** Up-regulated genes in both LNCaP^R^ and C4-2B^R^.

Gene Symbol	Log2 Ratio		mRNA Accession	Probe Set ID
	LNCaP^R^ vs. LNCaP	C4-2B^R^ vs. C4-2B		
NOSTRIN	4.1	2.71	NM_001171631	TC0900010056.hg.1
REKIMU	4.17	11.1	rekimu.aAug10-unspliced	TC0400010553.hg.1
TNFSF15	4.31	8.42	NM_001204344	TC0600007847.hg.1
FIRRE	17.9	2.36	NR_026975	TC1000010727.hg.1
ABCB1	10.3	20.1	NM_000927	TC0700011706.hg.1
SH3BGRL	7.9	1.8	NM_003022	TC0500013296.hg.1
RUNDC3B	174.8	107.4	NM_001134405	TC0400008183.hg.1
SLC25A40	3.97	2.28	NM_018843	TC1200012199.hg.1
TP53INP1	3.92	2.54	NM_001135733	TC1300008371.hg.1
RYR2	3.29	2.54	NM_001035	TC0500013298.hg.1
TM4SF1	3.5	41	NM_014220	TC0600012839.hg.1
CDK19	3.7	2.62	NM_001300960	TC0300011309.hg.1
CDKN1A	9.65	3.25	NM_000389	TC2100007821.hg.1
SLFN5	3.8	5.59	NM_144975	TC2000007943.hg.1
DUSP16	2.95	2.17	NM_030640	TC0500013297.hg.1

**Table 2:** Down-regulated genes in both LNCaP^R^ and C4-2B^R^.

Gene Symbol	Log2 Ratio		mRNA Accession	Probe Set ID
	LNCaP^R^ vs. LNCaP	C42B^R^ vs. C42B		
FAM72B	0.23	0.32	NM_001100910	TC0X00010826.hg.1
PTGFR	0.04	0.21	NM_000959	TC1500008627.hg.1
CENPJ	0.38	0.45	NM_018451	TC0500011648.hg.1
KIF20B	0.31	0.42	NM_001284259	TC1000008406.hg.1
LIN7A	0.24	0.09	NM_004664	TC1900008607.hg.1
CDH26	0.17	0.44	NM_021810	TSUnmapped00000374.hg.1
BEND4	0.14	0.31	NM_001159547	TC0100014543.hg.1
FAM72D	0.31	0.31	NM_207418	TC0700008181.hg.1
DEPDC1	0.04	0.24	NM_001114120	TC0100015509.hg.1
HIST1H1B	0.21	0.24	NM_005322	TC0600011232.hg.1
EFNA5	0.1	0.18	NM_001962	TC0100009723.hg.1
CHYSLOBY	0.17	0.22	chysloby.aAug10-unspliced	TC0400012245.hg.1
SKERSWORBY	0.24	0.06	skersworby.aAug10-unspliced	TC1800009011.hg.1
NMU	0.21	0.52	NM_001292045	TC1200011385.hg.1
ANKRD18A	0.14	0.44	NM_147195	TC0500011649.hg.1

## Data Availability

The data presented in this study are available on request from the corresponding author. The data are not publicly available due to restrictions on availability.

## Supplementary Materials

The following are available online at https://www.mdpi.com/2072-6694/13/6/1290/s1, Figure S1: Uncropped Western Blot from Figure 5a, Figure S2: Uncropped Western Blot from Figure 6a, Figure S3: Uncropped Western Blot from Figure 6d, Table S1: Significantly deregulated genes found in docetaxel-resistant C4-2B^R^ cells compared to matched parental C4-2B cells, Table S2: Significantly deregulated genes found in docetaxel-resistant LNCaP^R^ cells compared to the matched parental LNCaP cell line.
