# Supplementary material for: Molecular Profiling of Docetaxel-Resistant Prostate Cancer Cells Identifies Multiple Mechanisms of Therapeutic Resistance
Source: Cancers (Basel). 2021 Mar 14;13(6):1290. doi: 10.3390/cancers13061290 (PMC7998254; doi:10.3390/cancers13061290)
Supplement: Supplementary file 1 [file cancers-13-01290-s001.pdf]

Supplementary Material: Molecular Profiling of Docetaxel-Resistant Prostate Cancer Cells Identifies Multiple Mechanisms of Therapeutic Resistance

Tiago S. Lima, Diego Iglesias-Gato, Luciano D.O. Souza, Jan Stenvang, Diego S. Lima, Martin A. Røder, Klaus Brasso and José M.A. Moreira

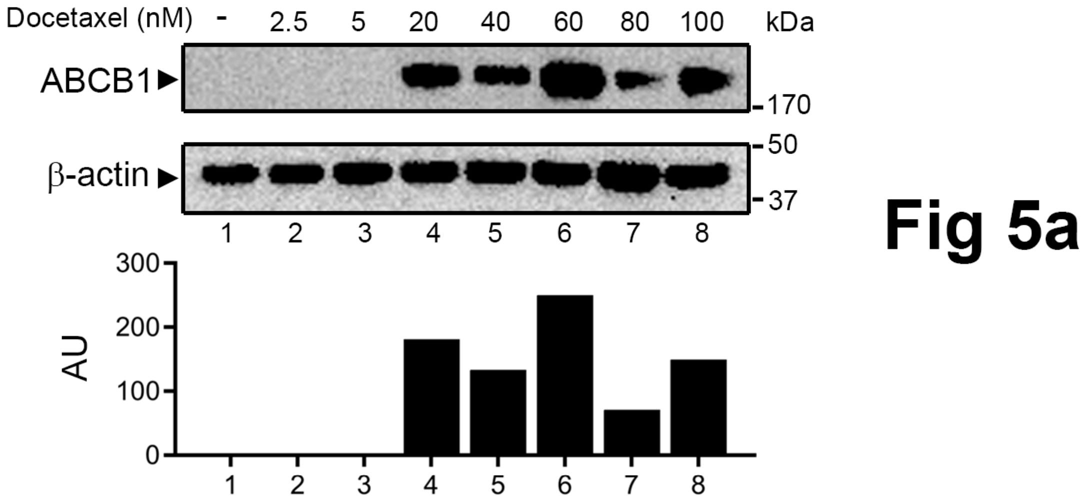

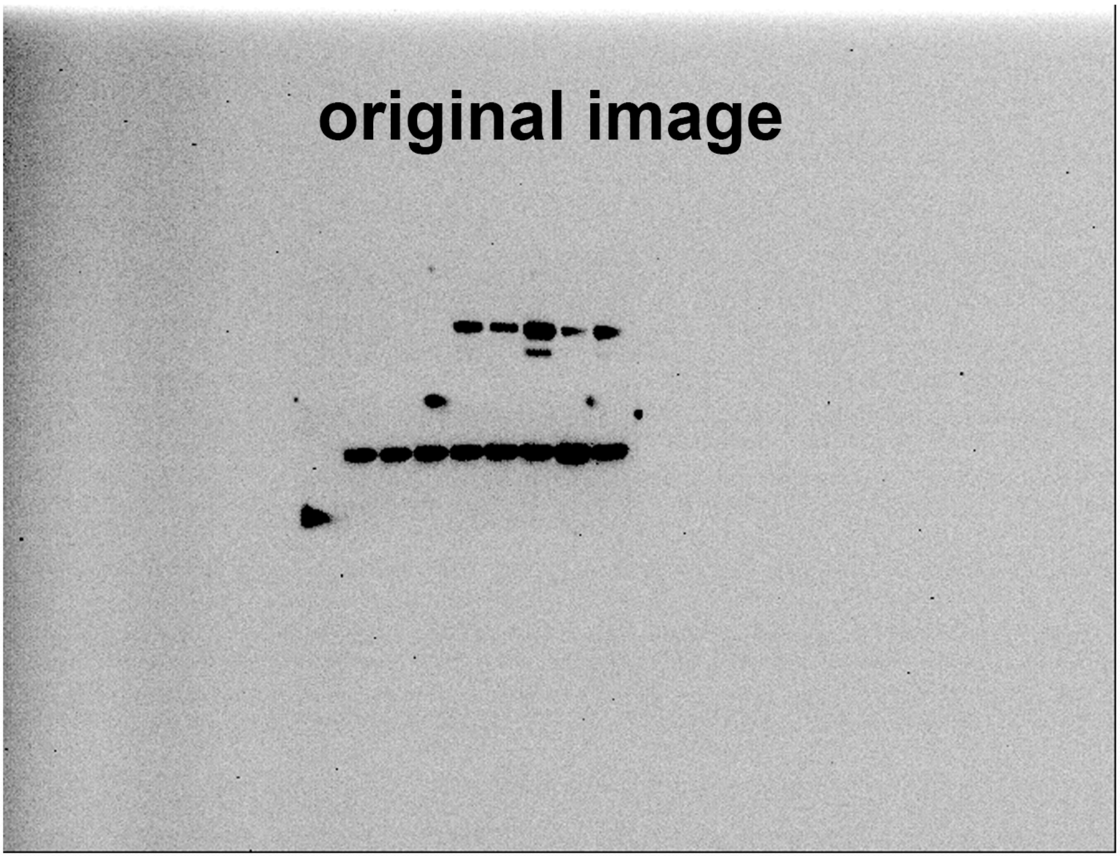

Figure S1. Uncropped Western Blot from Figure 5a.

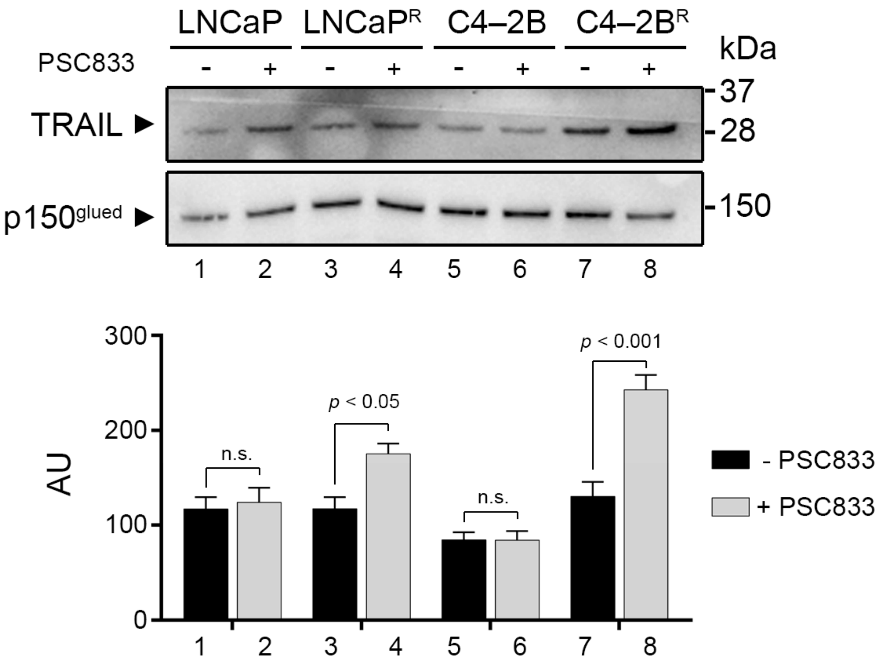

Fig 6a

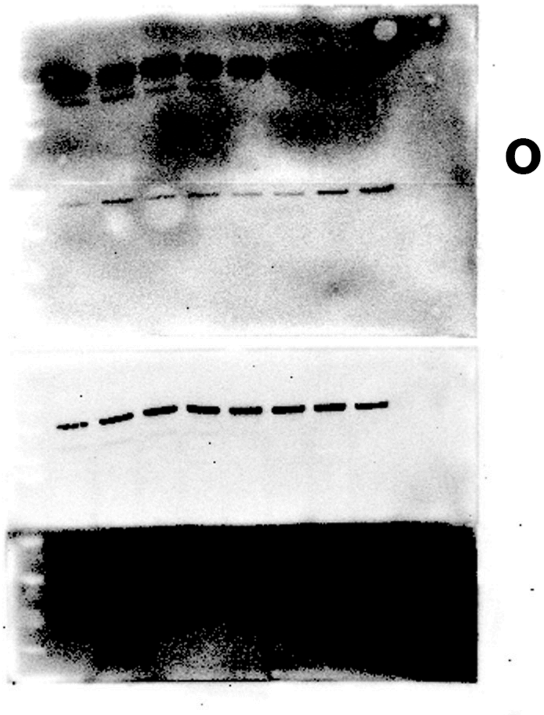

original images

Figure S2. Uncropped Western Blot from Figure 6a.

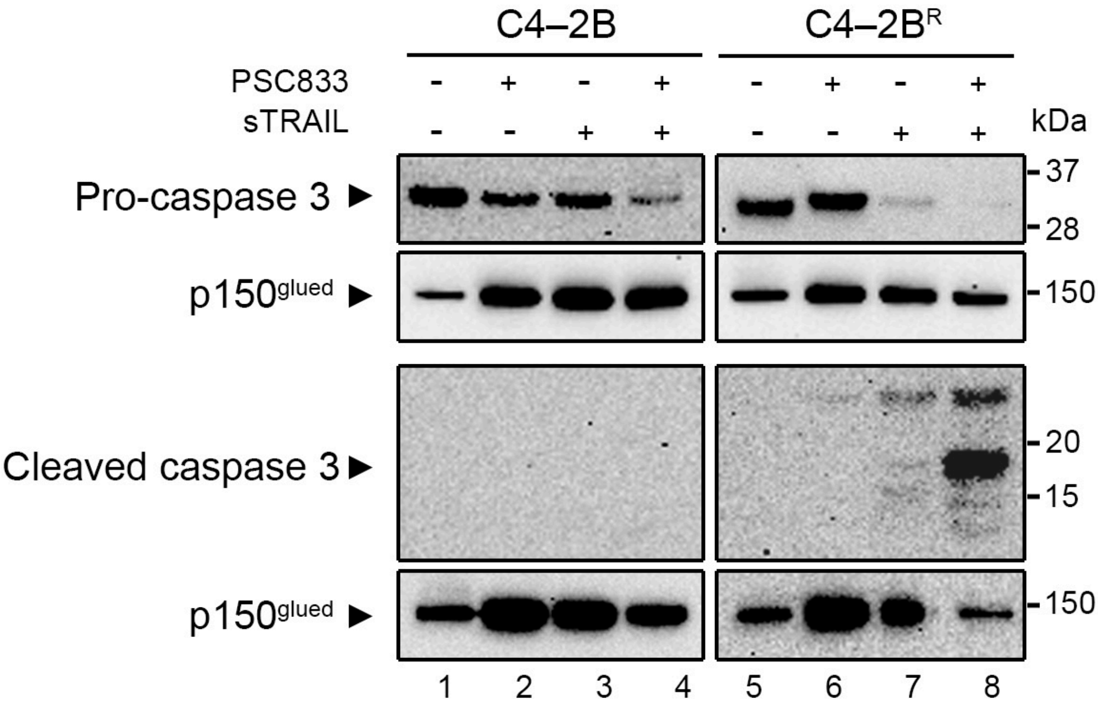

Fig 6d

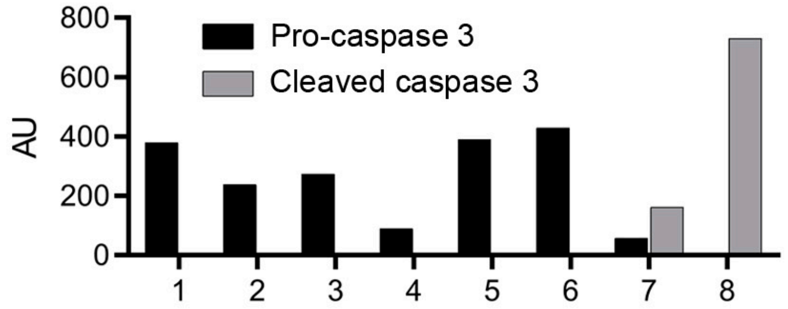

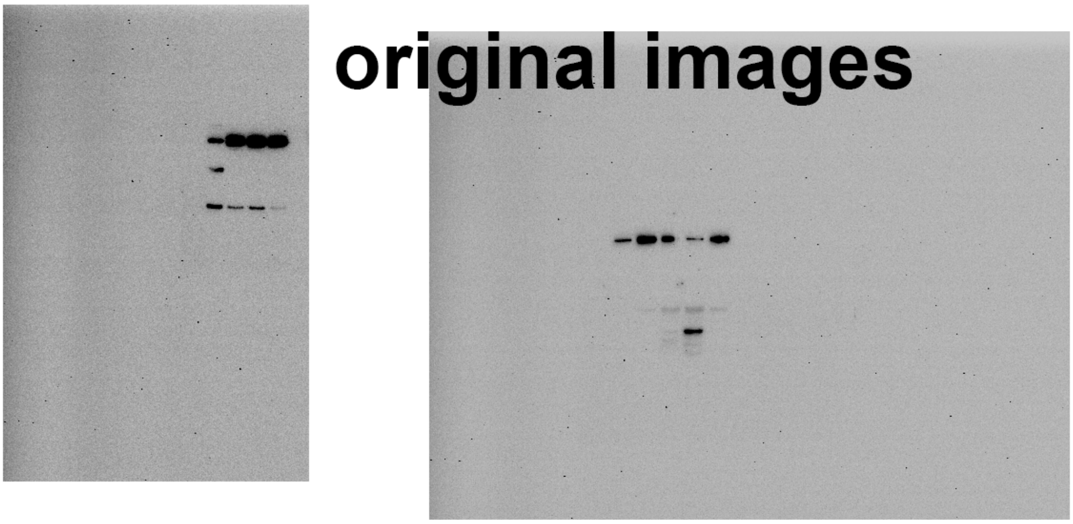

Figure S3. Uncropped Western Blot from Figure 6d.

Table 1. Significantly deregulated genes found in docetaxel resistant C4-2B[R] cells compared to matched parental C4-2B cells.

Gene Symbol	Log2 ratio C42B-R vs. C42B Parental	mRNA Accession	Probe Set ID
MTCO2P20	2.556207	ENST00000437339	TC2200007234.hg.1
sloytar	1.8353629	sloytar.aAug10-unspliced	TC1500008258.hg.1
FAM46C	1.9558613	NM_017709	TC0100009572.hg.1
MT1B	3.5956287	NM_005947	TC1600007964.hg.1
VCX2	1.8150125	NM_016378	TC0X00009022.hg.1
MYLK	2.063236	NM_053025	TC0300012212.hg.1
TRIM25	1.8256367	NM_005082	TC1700011208.hg.1
HYOU1	1.9994713	ENST00000629679	TSUnmapped00000322.hg.1
PLS1	2.0099387	NM_001145319	TC0300009033.hg.1
SLC25A40	2.2871358	NM_018843	TC0700011710.hg.1
DSTN	2.6777968	NM_001011546	TC2000006799.hg.1
flubu	3.4557462	flubu.aAug10-unspliced	TC0100016681.hg.1
WDR44	1.7549098	NM_001184965	TC0X00008223.hg.1
MEF2C	1.6524632	NM_001131005	TC0500011418.hg.1
DUX4	1.8980595	NM_001293798	TC0400012880.hg.1
KCNS3	1.9054757	NM_001282428	TC0200016408.hg.1
SMARCA2	1.8613421	NM_001289396	TC0900006473.hg.1
NOS3	1.8820606	NM_000603	TC0700009688.hg.1
snorrawby	2.5780115	snorrawby.aAug10-unspliced	TC0600012435.hg.1
FICD	2.2886028	NM_007076	TC1200012702.hg.1
C8orf4	1.7585952	NM_020130	TC0800007389.hg.1
AL158839.1	7.5121217	uc057hmg.1	TC0100014568.hg.1
FKBP14	2.336669	NM_017946	TC0700010621.hg.1
CCNG2	1.9747869	NM_004354	TC0400012818.hg.1
swola	2.1214652	swola.aAug10-unspliced	TC0800011605.hg.1
SH3BGRL2	1.8042784	NM_031469	TC0600008622.hg.1
RP11-175I6.1	2.4739854	ENST00000509595	TC0900010160.hg.1
PLEKHH2	2.2555783	NM_172069	TC0200007399.hg.1
VWA5B1	2.4121084	NM_001039500	TC0100007199.hg.1
SQSTM1	2.5798378	NM_001142298	TC0500009706.hg.1
HGD	2.4191759	NM_000187	TC0300012155.hg.1
HIST1H2BD	1.982365	NM_021063	TC0600007274.hg.1
PACSIN2	2.1343384	NM_001184970	TC2200009357.hg.1
KCNG1	2.3609717	NM_002237	TC2000009448.hg.1
PECR	1.8237522	NM_018441	TC0200016768.hg.1
spostybu	2.5164812	spostybu.aAug10-unspliced	TC0500010494.hg.1
CIR1P2	1.6461604	ENST00000514821	TC0400011658.hg.1
RP1-276J11.2	1.873519	ENST00000407291	TC0600009306.hg.1
SLPI	4.85093	NM_003064	TC2000009216.hg.1
snarsmer	1.6336102	snarsmer.aAug10-unspliced	TC2100008332.hg.1
beyswa	2.444127	beyswa.aAug10-unspliced	TC1500010226.hg.1
smarvey	2.0148034	smarvey.aAug10-unspliced	TC2000009625.hg.1
RP11-467M13.3	2.8945584	ENST00000563361	TC1600009569.hg.1
HIST1H2AC	2.0392265	NM_003512	TC0600007270.hg.1
nawsharby	2.0934248	nawsharby.aAug10-unspliced	TC0500008920.hg.1
EPB41	1.8033892	NM_001166005	TC0100007574.hg.1
BMP7	2.7241943	NM_001719	TC2000009579.hg.1
stawzawby	1.9640744	stawzawby.aAug10-unspliced	TC0800009618.hg.1
USP54	2.2468677	NM_152586	TC1000011026.hg.1
CRIPT	1.8644994	NM_014171	TC0200007473.hg.1
PHLDA1	2.250327	NM_007350	TC1200011310.hg.1

Gene Symbol	Log2 ratio C42B-R vs. C42B Parental	mRNA Accession	Probe Set ID
TMSB10P2	1.9637421	ENST00000416049	TC0X00006721.hg.1
ESRP1	1.9688116	NM_001034915	TC0800008263.hg.1
STK3	1.8991319	NM_001256312	TC0800011216.hg.1
skoytyby	1.9804734	skoytyby.aAug10-unspliced	TC0900011615.hg.1
U3	2.7957277	uc060hzu.1	TC1700011264.hg.1
beyforbo	1.8752613	beyforbo.aAug10-unspliced	TC0300012830.hg.1
OR7E108P	2.0346537	OTTHUMT00000469013	TC0900010741.hg.1
STXBP1	1.6425661	NM_001032221	TC0900008811.hg.1
NEK11	2.1769638	NM_001146003	TC0300008826.hg.1
RAB27B	2.273912	NM_004163	TC1800007360.hg.1
MGST1	2.129763	NM_001260511	TC1200012597.hg.1
FAM3B	2.6562383	NM_058186	TC2100007204.hg.1
FBXW7	1.7710307	NM_001013415	TC0400012143.hg.1
C10orf113	2.31503	NM_001010896	TC1000009985.hg.1
ANKRD30A	1.9212155	NM_052997	TC1000007305.hg.1
TCEA1	2.4389772	NM_006756	TC0800010458.hg.1
RPL10L	1.6362087	NM_080746	TC1400009074.hg.1
SNX24	2.4728703	NM_014035	TC0500008483.hg.1
LDOC1	3.1814852	NM_012317	TC0X00010989.hg.1
MT1L	2.7971764	NR_001447	TC1600007958.hg.1
RNF144B	2.8865757	NM_182757	TC0600007108.hg.1
APOL2	2.8770442	NM_030882	TC2200008592.hg.1
FAM102A	2.5191514	NM_001035254	TC0900011613.hg.1
HYOU1	2.0172553	ENST00000630070	TSUnmapped00000383.hg.1
NUDT4P1	2.0543	NR_002212	TC0100009760.hg.1
NUDT4	2.0543	BC012069	TC0100009833.hg.1
MT2P1	2.004661	ENST00000262499	TC0400007731.hg.1
SLC39A8	2.430937	NM_001135146	TC0400012949.hg.1
ABCA4	1.7557682	NM_000350	TC0100014977.hg.1
AFF1	2.1071823	NM_001166693	TC0400008053.hg.1
nihayu	2.299007	nihayu.aAug10-unspliced	TC0500013134.hg.1
LYPLAL1	1.997061	NM_001300769	TC0100018344.hg.1
styloyby	2.2864542	styloyby.aAug10-unspliced	TC1100012806.hg.1
MT1H	2.0951805	NM_005951	TC1600007966.hg.1
charskarbu	1.7623106	charskarbu.aAug10-unspliced	TC0800007359.hg.1
TFRC	3.0779295	NM_001128148	TC0300013684.hg.1
PBX1	2.1003077	NM_001204961	TC0100010416.hg.1
C11orf74	1.869461	NM_001276722	TC1100007307.hg.1
skoweyby	1.9326919	skoweyby.aAug10-unspliced	TC0800007025.hg.1
berpu	3.4157875	berpu.aAug10-unspliced	TC1600009568.hg.1
GAGE2D	1.9100043	NM_001098407	TC0X00007230.hg.1
AKR1C1	3.9698596	NM_001353	TC1000012427.hg.1
SDC4	2.9199095	NM_002999	TC2000009218.hg.1
CEBPG	2.0440154	NM_001252296	TC1900007777.hg.1
ANXA9	2.6036305	NM_003568	TC0100009934.hg.1
SLC7A2	2.4294286	NM_001008539	TC0800006869.hg.1
DPY19L2	2.7795074	NM_173812	TC1200011067.hg.1
LNX1	1.848864	NM_001126328	TC0400010704.hg.1
RP11-310H4.6	1.7799234	ENST00000432235	TC0700011150.hg.1
pumeybo	2.9632332	pumeybo.aAug10-unspliced	TC0200016307.hg.1
barter	3.1832414	barter.aAug10-unspliced	TC1900008897.hg.1
KIAA1217	2.0498295	NM_001098500	TC1000007024.hg.1
SCPEP1	2.0521278	NM_021626	TC1700008372.hg.1
norome	2.8114371	norome.aAug10-unspliced	TC0600007272.hg.1

Gene Symbol	Log2 ratio C42B-R vs. C42B Parental	mRNA Accession	Probe Set ID
GOLPH3L	1.9845945	NM_018178	TC0100015747.hg.1
GAGE12C	1.6847732	NM_001098408	TC0X00007236.hg.1
FIRRE	2.36869	NR_026975	TC0X00010826.hg.1
STOX2	2.7031202	NM_020225	TC0400012868.hg.1
TGFB2	1.9736983	NM_001135599	TC0100011621.hg.1
GFPT1	2.057595	NM_001244710	TC0200012956.hg.1
CCSER2	2.063996	NM_001284240	TC1000008276.hg.1
PITPNC1	2.7577353	NM_012417	TC1700008690.hg.1
TPM4	1.8586121	NM_001145160	TC1900007260.hg.1
FAM49A	2.2904944	NM_030797	TC0200011837.hg.1
UGT2B17	3.4642975	NM_001077	TC0400012923.hg.1
IFIH1	2.2537599	NM_022168	TC0200014772.hg.1
PRSS3P1	2.3051689	OTTHUMT00000352529	TC0700013482.hg.1
MIR5087	3.0674887	NR_049810	TC0100015651.hg.1
spokabu	2.7656844	spokabu.aAug10-unspliced	TC0100016834.hg.1
OCLN	1.9891542	NM_001205254	TC0500007681.hg.1
FTH1P20	2.416377	ENST00000434913	TC0200015115.hg.1
LYNX1	3.1316922	NM_023946	TC0800012087.hg.1
GIMAP2	2.6012378	NM_015660	TC0700009678.hg.1
OGT	2.440051	NM_181672	TC0X00007573.hg.1
BZW1	2.2101567	NM_001207067	TC0200016591.hg.1
SPATA6	2.8650463	NM_001286238	TC0100014127.hg.1
ADGRD1	1.9458314	NM_198827	TC1200009420.hg.1
NSFL1C	1.9769204	NM_001206736	TC2000009967.hg.1
HTRA1	3.3722675	NM_002775	TC1000009152.hg.1
RYR2	2.540142	NM_001035	TC0100012113.hg.1
PDZRN4	2.0251794	NM_001164595	TC1200007365.hg.1
TMPRSS13	2.5395088	NM_001077263	TC1100012460.hg.1
TGIF1	2.3427405	NM_001278682	TC1800006513.hg.1
CAPN13	2.8812177	NM_144575	TC0200012159.hg.1
DDIT3	2.8651576	NM_001195053	TC1200010968.hg.1
flageyby	2.9376454	flageyby.aAug10-unspliced	TC0800006607.hg.1
ARHGAP18	2.7072875	NM_033515	TC0600013147.hg.1
RP11-42I10.3	2.433308	ENST00000569247	TC1600010232.hg.1
AHNAK	2.3978548	NM_001620	TC1100011083.hg.1
SLC31A1	2.3184505	NM_001859	TC0900008483.hg.1
INSIG1	2.1990108	NM_005542	TC0700009807.hg.1
ZMAT1	2.786966	NM_001011657	TC0X00010375.hg.1
RASSF5	2.057347	NM_182663	TC0100011378.hg.1
CDKL5	2.6031282	NM_001037343	TC0X00006723.hg.1
SLC6A9	2.9733095	NM_001024845	TC0100013949.hg.1
NFE2L1	1.8720367	NM_003204	TC1700008151.hg.1
APOBEC3F	2.2555275	NM_001006666	TC2200009273.hg.1
FTH1P1	2.5797894	ENST00000437933	TC0100007836.hg.1
SLC27A2	2.3830538	NM_001159629	TC1500007196.hg.1
sporrer	2.0543048	sporrer.aAug10-unspliced	TC1700010884.hg.1
DNAJB4	1.7952117	NM_007034	TC0100018238.hg.1
MOCOS	2.0550597	NM_017947	TC1800007092.hg.1
SMAD1	1.9369818	NM_001003688	TC0400008909.hg.1
blufleyby	2.6883414	blufleyby.aAug10-unspliced	TC0600010924.hg.1
G6PD	2.6381767	NM_000402	TC0X00011211.hg.1
COX7B2	2.2448897	NM_130902	TC0400010601.hg.1
RNF24	2.4011421	NM_001134337	TC2000008242.hg.1
NOSTRIN	2.720015	NM_001171631	TC0200009902.hg.1

Gene Symbol	Log2 ratio C42B-R vs. C42B Parental	mRNA Accession	Probe Set ID
DUSP16	2.177465	ENST00000629698	TSUnmapped00000353.hg.1
ARL6IP5	2.2438483	NM_006407	TC0300007745.hg.1
SERINC2	3.0051534	NM_001199037	TC0100007638.hg.1
GAS6-AS1	2.4923854	NR_044995	TC1300010008.hg.1
KGFLP2	2.3089495	NR_003670	TC0900010077.hg.1
SCD	3.149445	NM_005063	TC1000008643.hg.1
CPEB4	2.6830664	NM_001308189	TC0500009521.hg.1
ZNF716	2.8257208	NM_001159279	TC0700007692.hg.1
ATXN1	3.0712938	NM_000332	TC0600010921.hg.1
GBP2	3.174421	NM_004120	TC0100018451.hg.1
MCUR1	2.685324	NM_001031713	TC0600010867.hg.1
FTH1P3	3.7188716	NR_002201	TC0200012078.hg.1
BCAT1	2.335521	NM_001178091	TC1200010145.hg.1
TGFBR3	3.277282	NM_001195683	TC0100014910.hg.1
ANKDD1A	2.2231696	NM_182703	TC1500010755.hg.1
SC5D	3.3064392	NM_001024956	TC1100009306.hg.1
HSPB1	2.580128	NM_001540	TC0700008090.hg.1
SLC41A2	2.5899184	NM_032148	TC1200011767.hg.1
TPD52L1	2.589391	NM_001003395	TC0600009353.hg.1
MPZL2	2.4309084	NM_005797	TC1100012474.hg.1
EBAG9	1.9143658	NM_001278938	TC0800008550.hg.1
PARP14	3.007323	NM_017554	TC0300008561.hg.1
PHLDA2	3.3740947	NM_003311	TC1100009817.hg.1
GAGE2B	2.1062987	NM_001098411	TC0X00011289.hg.1
TUFT1	1.8593719	NM_001126337	TC0100009959.hg.1
SESN2	3.0843472	NM_031459	TC0100007552.hg.1
SPTLC3	4.023873	NM_018327	TC2000006736.hg.1
CAST	2.2886567	NM_001042440	TC0500013204.hg.1
SRD5A3	3.0292084	NM_024592	TC0400007556.hg.1
MTND2P19	1.9594035	ENST00000449129	TC0200014466.hg.1
RP11-262H14.11	1.9232662	ENST00000402994	TC0900007367.hg.1
RANBP9	2.251033	NM_005493	TC0600010864.hg.1
RNVU1-14	2.1135805	NR_104075	TC0100009731.hg.1
CAPN2	2.5348122	NM_001146068	TC0100011721.hg.1
UGT2B15	4.087487	NM_001076	TC0400012924.hg.1
SP140L	3.761476	NM_001308162	TC0200011022.hg.1
ANK2	3.0075157	NM_001127493	TC0400008450.hg.1
NUDT4	2.2091818	NM_001301022	TC1200008466.hg.1
PRSS3	2.397254	NM_001197097	TC0900007006.hg.1
sakaya	5.2009363	sakaya.aAug10-unspliced	TC0600010934.hg.1
CA12	3.5536072	NM_001218	TC1500009709.hg.1
MAP4	2.3073993	NM_001134364	TC0300010999.hg.1
RTN3	3.4215872	NM_001265589	TC1100007906.hg.1
CDH1	2.870005	NM_004360	TC1600008209.hg.1
RPL10P5	2.1095893	ENST00000427719	TC0200013812.hg.1
FTH1P5	2.7968874	ENST00000425761	TC0600012024.hg.1
SHTN1	2.751711	NM_001127211	TC1000011938.hg.1
FYN	1.9693853	NM_002037	TC0600012875.hg.1
GBA3	2.4332054	NM_001128432	TC0400007054.hg.1
DIAPH2	2.2851849	NM_006729	TC0X00007889.hg.1
SEC24D	1.9789906	NM_014822	TC0400011695.hg.1
CSTA	2.761778	NM_005213	TC0300008550.hg.1
TMEM2	3.2893984	NM_001135820	TC0900010386.hg.1
ZNRF2	2.6592278	NM_147128	TC0700013343.hg.1

Gene Symbol	Log2 ratio C42B-R vs. C42B Parental	mRNA Accession	Probe Set ID
HMGCS1	2.1856532	NM_001098272	TC0500010635.hg.1
MYL6	1.9070586	NM_021019	TC1200012648.hg.1
SEMA3C	3.134626	NM_006379	TC0700011626.hg.1
ELOVL6	7.713397	NM_001130721	TC0400012956.hg.1
STAT1	2.125659	NM_007315	TC0200015242.hg.1
smeyneeby	3.4638515	smeyneeby.aAug10-unspliced	TC1000006960.hg.1
FGF7P2	2.568896	OTTHUMT00000157659	TC2100006546.hg.1
EPHX1	2.696237	NM_000120	TC0100011770.hg.1
GRIN3A	2.0824683	NM_133445	TC0900011038.hg.1
garjer	6.5080347	garjer.aAug10-unspliced	TC1900010559.hg.1
foyjer	6.5080347	foyjer.aAug10-unspliced	TC1900010557.hg.1
CASP10	3.0343173	NM_001206524	TC0200010445.hg.1
FKBP1A-SDCBP2	4.099933	NR_037661	TC2000009966.hg.1
FIBIN	3.2078543	NM_203371	TC1100007110.hg.1
AMD1	2.3803315	NM_001287214	TC0600009127.hg.1
CD58	3.0199096	NM_001144822	TC0100015397.hg.1
tuhiyo	2.0951114	tuhiyo.aAug10-unspliced	TC0200010130.hg.1
LAMA3	2.606424	NM_000227	TC1800006897.hg.1
MT1X	2.7078235	NM_005952	TC1600011399.hg.1
RP11-164A6.1	3.8277433	ENST00000566263	TC1600007067.hg.1
ADAM22	3.3546476	NM_004194	TC0700008265.hg.1
CTAGE5	2.3504632	NM_001247988	TC1400010605.hg.1
HSPB8	5.3794823	NM_014365	TC1200012714.hg.1
SEPHS2	1.8761007	NM_012248	TC1600009967.hg.1
CDRT1	2.8424735	NM_001282540	TC1700012361.hg.1
AGMO	5.1053877	NM_001004320	TC0700010334.hg.1
SERPINA3	3.7901878	NM_001085	TC1400010644.hg.1
GPR1	4.6435003	NM_001098199	TC0200015514.hg.1
ESYT1	3.766751	NM_001184796	TC1200012646.hg.1
TRIM16L	2.9274688	NM_001037330	TC1700007135.hg.1
MAP1LC3B	3.7100227	NM_022818	TC1600011442.hg.1
TBL1X	2.7703466	NM_001139466	TC0X00006581.hg.1
CDKN1A	3.2562292	NM_000389	TC0600007847.hg.1
TLR3	2.6054356	NM_003265	TC0400009543.hg.1
MVB12A	2.9125915	NM_001304547	TC1900007325.hg.1
FBXL2	2.5896528	NM_001171713	TC0300006994.hg.1
ABCB4	2.3626509	NM_000443	TC0700011705.hg.1
TP53INP1	2.546145	NM_001135733	TC0800011150.hg.1
VOPP1	2.7426176	NM_001284282	TC0700011139.hg.1
SH3BGRL	3.8019726	NM_003022	TC0X00007744.hg.1
HSPB1P1	3.0172966	ENST00000423240	TC0900010395.hg.1
CCPG1	2.8027964	NM_001204450	TC1500010869.hg.1
RCN1	2.0356863	NM_002901	TC1100012981.hg.1
MT2A	3.3000278	NM_005953	TC1600007957.hg.1
sugorby	2.5419974	sugorby.aAug10-unspliced	TC0800006629.hg.1
TES	2.4288614	NM_015641	TC0700008868.hg.1
CRNDE	2.7284465	NM_001308963	TC1600010375.hg.1
SLC18B1	2.812597	NM_052831	TC0600013206.hg.1
SYTL2	3.361905	NM_001162951	TC1100011833.hg.1
ERAP1	2.9046552	NM_001040458	TC0500013342.hg.1
ITGAV	1.9961687	NM_001144999	TC0200010216.hg.1
DEPTOR	3.7637937	NM_001283012	TC0800008674.hg.1
XAGE1B	2.9679797	NM_001097594	TC0X00007285.hg.1
BTN3A3	2.6942353	NM_001242803	TC0600007306.hg.1

Gene Symbol	Log2 ratio C42B-R vs. C42B Parental	mRNA Accession	Probe Set ID
NQO1	3.478722	NM_000903	TC1600010732.hg.1
EFHC2	3.9557226	NM_025184	TC0X00009493.hg.1
EGF	2.5789373	NM_001178130	TC0400008391.hg.1
S100A11	3.4654043	NM_005620	TC0100015822.hg.1
SVIL	2.0556378	NM_003174	TC1000010172.hg.1
IYD	3.2605581	NM_001164694	TC0600009836.hg.1
SORL1	3.5583835	NM_003105	TC1100009309.hg.1
ITGA6	3.3327804	NM_000210	TC0200009973.hg.1
GOLIM4	2.9543908	NM_001308155	TC0300013058.hg.1
COL5A1	2.6093705	NM_000093	TC0900009127.hg.1
NLRP11	5.0331736	NM_001297743	TC1900011495.hg.1
NMRK1	3.1615076	NM_001127603	TC0900010445.hg.1
CD47	3.0799217	NM_001777	TC0300011936.hg.1
CYP2R1	2.9326434	NM_024514	TC1100010185.hg.1
FAM3C	2.5577636	NM_001040020	TC0700012427.hg.1
SEL1L3	3.334759	NM_001297592	TC0400010282.hg.1
NBR2	2.5912611	NR_003108	TC1700007939.hg.1
BTN3A2	5.676165	NM_001197246	TC0600007301.hg.1
SCARB2	2.6410038	NM_001204255	TC0400011057.hg.1
MATN2	2.6245186	NM_002380	TC0800008324.hg.1
C11orf1	2.9532487	NM_022761	TC1100009040.hg.1
SLFN5	5.592034	NM_144975	TC1700007585.hg.1
SLC9A1	2.7157364	NM_003047	TC0100013417.hg.1
IDH1	2.9535055	NM_001282386	TC0200015578.hg.1
TACC1	3.6506128	NM_001122824	TC0800007351.hg.1
LINC01260	5.8145905	NR_034104	TC2000009199.hg.1
REXO2	4.1899905	NM_015523	TC1100013097.hg.1
TXNRD1	2.734969	NM_001093771	TC1200008675.hg.1
STX3	3.2840781	NM_001178040	TC1100013012.hg.1
TRPV6	4.233062	NM_018646	TC0700012867.hg.1
CLCN3P1	2.7573285	ENST00000423965	TC0900012207.hg.1
IFT46	3.3162656	NM_001168618	TC1100012488.hg.1
skumu	6.3857255	skumu.aAug10-unspliced	TC1900007926.hg.1
bawvy	4.0946937	bawvy.aAug10-unspliced	TC1900010558.hg.1
RNF43	2.7926848	NM_001305544	TC1700012439.hg.1
XAGE1E	3.3516333	NM_001097604	TC0X00009718.hg.1
muchee	4.837899	muchee.aAug10-unspliced	TC1900007965.hg.1
stawmo	4.837899	stawmo.aAug10-unspliced	TC1900007966.hg.1
LMO4	4.990986	NM_006769	TC0100008938.hg.1
TRAF5	2.2844217	NM_001033910	TC0100011487.hg.1
PIGA	2.6435392	NM_002641	TC0X00009124.hg.1
SLC5A1	7.196634	NM_000343	TC2200007120.hg.1
SOHLH2	5.655156	NM_001282147	TC1300010032.hg.1
ATP2B1	4.5906506	NM_001001323	TC1200011474.hg.1
MSMO1	3.3272045	NM_001017369	TC0400009221.hg.1
AGR2	7.5411777	NM_006408	TC0700010355.hg.1
CCDC169-SOHLH2	4.812886	NM_001198910	TC1300010031.hg.1
ARHGEF2	4.1870937	NM_001162383	TC0100018507.hg.1
CDK19	2.6237354	NM_001300960	TC0600012839.hg.1
SLC16A1	4.5411086	ENST00000629508	TSUnmapped00000372.hg.1
IFRD1	2.8767796	NM_001007245	TC0700013441.hg.1
IRF6	3.238593	NM_001206696	TC0100017172.hg.1
NPC1	2.7375877	NM_000271	TC1800008285.hg.1
ENAH	3.6579103	NM_001008493	TC0100017491.hg.1

Gene Symbol	Log2 ratio C42B-R vs. C42B Parental	mRNA Accession	Probe Set ID
RTN4	4.4425783	NM_007008	TC0200016672.hg.1
SDC2	2.9694257	NM_002998	TC0800008299.hg.1
NAALADL2	5.1968737	NM_207015	TC0300013904.hg.1
CA1	6.0031576	NM_001128829	TC0800010990.hg.1
SLC16A1	6.8362164	ENST00000629920	TSUnmapped00000370.hg.1
ATF3	6.918365	NM_001030287	TC0100011533.hg.1
GCNT3	7.1341777	NM_004751	TC1500007409.hg.1
SLC16A1	4.7119775	ENST00000628110	TSUnmapped00000474.hg.1
TNFSF15	8.423518	NM_001204344	TC0900011300.hg.1
IL1RN	3.9677577	NM_000577	TC0200016511.hg.1
SERPINI1	6.7106404	NM_001122752	TC0300009412.hg.1
PRKAR2B	2.997872	NM_002736	TC0700008745.hg.1
FDFT1	3.5051198	NM_001287742	TC0800006760.hg.1
SAGE1	3.769296	NM_018666	TC0X00008518.hg.1
CCR2	4.198822	NM_001123041	TC0300007256.hg.1
JUN	6.7944	NM_002228	TC0100014349.hg.1
TMEM154	5.604867	NM_152680	TC0400012150.hg.1
MFSD6	5.952086	NM_017694	TC0200010264.hg.1
MANBA	5.6267633	NM_005908	TC0400011464.hg.1
C1orf158	13,972065	NM_152290	TC0100006905.hg.1
XG	8.097232	NM_001141919	TC0X00006480.hg.1
PAX9	3.197338	NM_006194	TC1400006933.hg.1
KIAA1841	5.085828	NM_001129993	TC0200016454.hg.1
FTH1P7	3.222549	ENST00000450676	TC1300008309.hg.1
glormu	12.776982	glormu.aAug10-unspliced	TC1900007923.hg.1
blymu	12.776982	blymu.aAug10-unspliced	TC1900007920.hg.1
blermu	12.776982	blermu.aAug10-unspliced	TC1900007919.hg.1
pleemu	12.776982	pleemu.aAug10-unspliced	TC1900007929.hg.1
glumu	12.776982	glumu.aAug10-unspliced	TC1900007924.hg.1
plumu	12.776982	plumu.aAug10-unspliced	TC1900007930.hg.1
FKBP1A	3.864848	NM_000801	TC2000009965.hg.1
FTH1P8	4.889244	ENST00000498161	TC0X00008672.hg.1
ISCU	3.1932976	NM_001301140	TC1200008771.hg.1
GPNMB	4.448002	NM_001005340	TC0700006913.hg.1
IFI16	6.2759724	NM_001206567	TC0100010244.hg.1
rekimu	11.182986	rekimu.aAug10-unspliced	TC0700011707.hg.1
swoyneeby	5.683965	swoyneeby.aAug10-unspliced	TC1000009987.hg.1
PRSS1	8.865776	ENST00000311737	TC0700013481.hg.1
LPIN1	3.8061435	NM_001261427	TC0200016403.hg.1
TMEM123	4.1524496	NM_052932	TC1100012121.hg.1
CT45A9	12.90071	NM_001291540	TC0X00010918.hg.1
CT45A8	12.90071	NM_001291535	TC0X00010917.hg.1
QKI	4.03818	NM_001301085	TC0600010121.hg.1
FAM129A	5.8426924	NM_052966	TC0100016678.hg.1
machee	12.880194	machee.aAug10-unspliced	TC1900010561.hg.1
choserbu	15.196476	choserbu.aAug10-unspliced	TC1900007921.hg.1
klarmu	15.196476	klarmu.aAug10-unspliced	TC1900007925.hg.1
slumu	15.196476	slumu.aAug10-unspliced	TC1900007931.hg.1
narva	15.196476	narva.aAug10-unspliced	TC1900007927.hg.1
CT45A1	6.4254484	NM_001017417	TC0X00008516.hg.1
BCAP29	4.129672	NM_001008405	TC0700013439.hg.1
GCLM	5.316095	NM_001308253	TC0100014966.hg.1
kawjer	17.038025	kawjer.aAug10-unspliced	TC1900010563.hg.1
rawsarbo	17.038025	rawsarbo.aAug10-unspliced	TC1900010556.hg.1

Gene Symbol	Log2 ratio C42B-R vs. C42B Parental	mRNA Accession	Probe Set ID
geyjer	17.038025	geyjer.aAug10-unspliced	TC1900010560.hg.1
kajer	17.038025	kajer.aAug10-unspliced	TC1900010562.hg.1
CLCN3	4.78516	NM_001243372	TC0400009284.hg.1
UNC13C	6.261909	NM_001080534	TC1500007294.hg.1
P4HA2	4.6525226	NM_001017973	TC0500013354.hg.1
CT45A10	13,91298	NM_001291527	TC0X00010919.hg.1
ERICH2	6.9932623	NM_001289947	TC0200009933.hg.1
LAMP3	21.869093	NM_014398	TC0300013336.hg.1
CT45A2	11.4338665	NM_152582	TC0X00010915.hg.1
CASP8	5.102216	NM_001080124	TC0200010447.hg.1
CT45A6	7.2247996	NM_001017438	TC0X00010916.hg.1
neeva	20.764526	neeva.aAug10-unspliced	TC1900007928.hg.1
HEXB	4.197721	NM_000521	TC0500007792.hg.1
CLDN12	4.0508637	NM_001185072	TC0700013396.hg.1
glermu	18,37179	glermu.aAug10-unspliced	TC1900007922.hg.1
CDR1	21.426937	NM_004065	TC0X00010982.hg.1
TSPAN7	5.3132873	NM_004615	TC0X00011279.hg.1
CASP4	10.432205	NM_001225	TC1100012165.hg.1
CT45A5	8.474921	NM_001007551	TC0X00010912.hg.1
PLA2G2A	17.562618	NM_000300	TC0100013162.hg.1
BHLHE41	6.6517124	NM_030762	TC1200010182.hg.1
GNAT3	8.211151	NM_001102386	TC0700011624.hg.1
BST2	18.39729	NM_004335	TC1900009970.hg.1
GNAI1	7.716185	NM_001256414	TC0700008181.hg.1
CT45A3	8.514448	NM_001017435	TC0X00010910.hg.1
ENTPD3	8.324273	NM_001248	TC0300007117.hg.1
ALDH3A2	7.4829698	NM_000382	TC1700007189.hg.1
PPIC	4.463586	NM_000943	TC0500011875.hg.1
GTPBP2	7.0732126	NM_001286216	TC0600011880.hg.1
stawvee	22,592394	stawvee.aAug10-unspliced	TC2100007844.hg.1
ID2	7.4938555	NM_002166	TC0200006627.hg.1
ENC1	7.352578	NM_001256574	TC0500011146.hg.1
CT45A7	9.232579	NM_001291543	TC0X00010914.hg.1
SLC16A1	10.68156	NM_001166496	TC0100015310.hg.1
ME1	5.6458917	NM_002395	TC0600012434.hg.1
SDCBP2	15.830707	NM_001199784	TC2000009964.hg.1
PTPDC1	7.2160754	NM_001253829	TC0900008010.hg.1
GABARAPL1	11,915585	NM_031412	TC1200006787.hg.1
IGFBP3	65.72888	NM_000598	TC0700010965.hg.1
VTRNA1-2	20.80613	NR_026704	TC0500008868.hg.1
ABCB1	20,164732	NM_000927	TC0700011706.hg.1
CLU	30.658182	NM_001831	TC0800009961.hg.1
MBNL2	29.695538	NM_001306070	TC1300007774.hg.1
VTRNA1-1	139.27454	NR_026703	TC0500008867.hg.1
TM4SF1	41.006676	NM_014220	TC0300012773.hg.1
RUNDC3B	107,40425	NM_001134405	TC0700008261.hg.1
AMACR	0.059065506	NM_001167595	TC0500013297.hg.1
C1QTNF3-AMACR	0.063421085	NR_037951	TC0500013296.hg.1
NCAM2	0.015936414	NM_004540	TC2100006714.hg.1
PRAC1	0.17386907	NM_032391	TC1700011002.hg.1
TNFRSF19	0.0226596	NM_001204458	TC1300006594.hg.1
COLCA1	0.05934625	NM_001302644	TC1100012285.hg.1
COL12A1	0.049540885	NM_004370	TC0600012314.hg.1
HMGN2P46	0.106946535	NR_022014	TC1500010735.hg.1

Gene Symbol	Log2 ratio C42B-R vs. C42B Parental	mRNA Accession	Probe Set ID
EIF1AY	0.06992707	NM_001278612	TC0Y00006730.hg.1
NETO1	0.06653976	NM_001201465	TC1800009011.hg.1
SSFA2	0.061340563	NM_001130445	TC0200010164.hg.1
soypoy	0.08891609	soypoy.aAug10-unspliced	TC1500008487.hg.1
USP9Y	0.07599683	NM_004654	TC0Y00006629.hg.1
LIN7A	0.09255946	NM_004664	TC1200011385.hg.1
DDX3Y	0.036294784	NM_001122665	TC0Y00006632.hg.1
EPHA6	0.057879977	NM_001080448	TC0300008082.hg.1
SLC30A4	0.045821954	NM_013309	TC1500009298.hg.1
NOVA1	0.1565771	NM_002515	TC1400008780.hg.1
ELOVL7	0.049077235	NM_001104558	TC0500010869.hg.1
ABCC4	0.07541325	NM_001105515	TC1300009505.hg.1
CHPT1	0.118322596	NM_020244	TC1200012698.hg.1
GPR158	0.11042797	NM_020752	TC1000007061.hg.1
PVRL3	0.10431223	NM_001243286	TC0300008316.hg.1
IGF1R	0.03335627	NM_000875	TC1500008485.hg.1
snarlyby	0.12991005	snarlyby.aAug10-unspliced	TC1500008499.hg.1
RPS4Y1	0.02991805	NM_001008	TC0Y00007322.hg.1
MIPEP	0.13367724	NM_005932	TC1300008338.hg.1
SYCP3	0.20759073	NM_001177948	TC1200011710.hg.1
tupoy	0.23465252	tupoy.aAug10-unspliced	TC1500010596.hg.1
FAM198B	0.0953856	NM_001031700	TC0400012245.hg.1
MANEA	0.13947152	NM_024641	TC0600008863.hg.1
skersworby	0.06479586	skersworby.aAug10-unspliced	TC0300008083.hg.1
NBEAP1	0.17064168	NR_027992	TC1500008627.hg.1
TENM1	0.13144097	NM_001163278	TC0X00010734.hg.1
SLC45A3	0.054192107	NM_033102	TC0100017073.hg.1
KCNN2	0.100247286	NM_001278204	TC0500008356.hg.1
SMAD5-AS1_2	0.12412887	ENST00000625970	TC0X00010737.hg.1
GCNT1	0.12824103	NM_001097633	TC0900012141.hg.1
DLGAP5	0.13707192	NM_001146015	TC1400009248.hg.1
ODC1	0.24383058	NM_001287188	TC0200011720.hg.1
slorzu	0.07500562	slorzu.aAug10-unspliced	TC1500007124.hg.1
MPC2	0.2423454	NM_001143674	TC0100016318.hg.1
FXYD3	0.11792929	NM_001136007	TC1900007836.hg.1
PLK1	0.1562279	NM_005030	TC1600007235.hg.1
RP11-1084J3.1	0.14328061	ENST00000504847	TC0500007098.hg.1
HMGN2P46	0.12467633	ENST00000568669	TC1500007126.hg.1
RP11-312J18.5	0.2535122	ENST00000425270	TC0100010319.hg.1
MICAL1	0.19511811	NM_001159291	TC0600014333.hg.1
FAM162A	0.24056706	NM_014367	TC0300008551.hg.1
RP11-566K19.8	0.15390322	ENST00000616872	TC1500006504.hg.1
SGK3	0.3514962	NM_013257	TC0800012312.hg.1
spawma	0.045882825	spawma.aAug10-unspliced	TC2100006717.hg.1
GRB10	0.1746415	NM_001001549	TC0700011054.hg.1
ARG2	0.13277169	NM_001172	TC1400007500.hg.1
PNMA1	0.12000802	NM_006029	TC1400009668.hg.1
NUSAP1	0.18117537	NM_001243142	TC1500006999.hg.1
RHOU	0.21054453	NM_021205	TC0100018355.hg.1
PRMT6	0.27755216	NM_018137	TC0100009305.hg.1
RP11-467L19.11	0.22862989	ENST00000553844	TC1500008624.hg.1
SACS	0.16955183	NM_001278055	TC1300008329.hg.1
ELK4	0.20709777	NM_001973	TC0100017072.hg.1
PLK2	0.17837898	NM_001252226	TC0500010835.hg.1

Gene Symbol	Log2 ratio C42B-R vs. C42B Parental	mRNA Accession	Probe Set ID
CKB	0.1957353	NM_001823	TC1400010317.hg.1
RBM24	0.25934535	NM_001143941	TC0600007084.hg.1
FAM105A	0.15613861	NM_019018	TC0500006816.hg.1
bloteeby	0.2365744	bloteeby.aAug10-unspliced	TC0900010471.hg.1
DUSP4	0.11050549	NM_001394	TC0800010002.hg.1
PLEKHH1	0.13054936	NM_020715	TC1400007495.hg.1
BLOC1S6	0.21769437	NM_001311255	TC1500010736.hg.1
FANCI	0.23741315	NM_001113378	TC1500008252.hg.1
PMEPA1	0.11039992	NM_001255976	TC2000009604.hg.1
FAM83D	0.15709096	NM_030919	TC2000007341.hg.1
DLX1	0.2682124	NM_001038493	TC0200009967.hg.1
RBM3	0.15596639	NM_006743	TC0X00007195.hg.1
SPDEF	0.18106687	NM_001252294	TC0600011590.hg.1
CDK1	0.15799786	NM_001170406	TC1000007748.hg.1
KIF14	0.24324186	NM_001305792	TC0100016891.hg.1
AADAT	0.15300687	NM_001286682	TC0400012396.hg.1
UAP1	0.31822258	NM_003115	TC0100010381.hg.1
MIPOL1	0.2759439	NM_001195296	TC1400006943.hg.1
GNPNAT1	0.23473144	NM_198066	TC1400009190.hg.1
bawver	0.17049104	bawver.aAug10-unspliced	TC2000009606.hg.1
CCNA2	0.37050804	NM_001237	TC0400011748.hg.1
TOP2A	0.1720557	NM_001067	TC1700010618.hg.1
LRIG1	0.11914799	ENST00000631853	TSUnmapped00000527.hg.1
DSG2	0.18742837	NM_001943	TC1800007014.hg.1
UBE2E3	0.30076867	NM_001278554	TC0200010152.hg.1
lerchar	0.2866356	lerchar.aAug10-unspliced	TC1200011312.hg.1
HNRNPF	0.35788873	NM_001098204	TC1000010438.hg.1
LEO1	0.32642314	NM_001286430	TC1500009446.hg.1
NUP58	0.2296493	NM_001008564	TC1300006641.hg.1
CCNB2	0.12473247	NM_004701	TC1500007392.hg.1
MCTP2	0.20682152	NM_001159643	TC1500010792.hg.1
C1QTNF9B-AS1	0.27327463	NM_001014442	TC1300006599.hg.1
PDS5B	0.3102115	NM_015032	TC1300006810.hg.1
EFNA5	0.18636826	NM_001962	TC0500011648.hg.1
CDKN3	0.2098086	NM_001130851	TC1400007201.hg.1
CENPU	0.25246412	NM_024629	TC0400012620.hg.1
FAM214A	0.25739858	NM_001286495	TC1500009460.hg.1
MBOAT2	0.22546573	NM_138799	TC0200016626.hg.1
MMP16	0.15111978	NM_005941	TC0800011041.hg.1
DEGS1	0.27528557	NM_003676	TC0100011736.hg.1
HSBP1L1	0.23926227	NM_001136180	TC1800007829.hg.1
RFC3	0.29012954	NM_002915	TC1300006847.hg.1
KCNH8	0.18288943	NM_144633	TC0300006781.hg.1
HOMER2	0.31101528	NM_004839	TC1500010251.hg.1
ST7	0.23107894	NM_018412	TC0700008882.hg.1
FKBP5	0.18159579	NM_001145775	TC0600011635.hg.1
ARHGAP11A	0.2558602	ENST00000633634	TSUnmapped00000695.hg.1
TSTA3	0.24702784	NM_003313	TC0800012147.hg.1
TOX3	0.26068604	NM_001080430	TC1600010316.hg.1
RP11-32B5.2	0.28276327	ENST00000555285	TC1500008647.hg.1
KIF2C	0.1590539	NM_001297655	TC0100008101.hg.1
OSBPL8	0.2552854	NM_001003712	TC1200011321.hg.1
SETBP1	0.27496424	NM_001130110	TC1800007186.hg.1
DEPDC1	0.24050532	NM_001114120	TC0100014543.hg.1

Gene Symbol	Log2 ratio C42B-R vs. C42B Parental	mRNA Accession	Probe Set ID
LGALS8	0.32247704	NM_006499	TC0100012097.hg.1
PRC1	0.1779887	NM_001267580	TC1500010463.hg.1
OGDH	0.38713583	NM_001003941	TC0700007392.hg.1
TTK	0.20442137	NM_001166691	TC0600008630.hg.1
ALDH1A3	0.4241392	NM_000693	TC1500010800.hg.1
SMARCD2	0.28894892	NM_001098426	TC1700011424.hg.1
glaskaw	0.20407212	glaskaw.aAug10-unspliced	TC1500008488.hg.1
NRIP1	0.18133456	NM_003489	TC2100008545.hg.1
FAM135A	0.36547872	NM_001105531	TC0600008468.hg.1
NDC80	0.1921163	NM_006101	TC1800006484.hg.1
SMC4	0.31495026	NM_001002800	TC0300009337.hg.1
NLGN4Y	0.22973306	NM_001164238	TC0Y00006651.hg.1
mureha	0.20594318	mureha.aAug10-unspliced	TC0100009308.hg.1
snorgoyby	0.3110182	snorgoyby.aAug10-unspliced	TC1200009073.hg.1
torpoy	0.16098881	torpoy.aAug10-unspliced	TC1500008495.hg.1
SLC39A10	0.36415023	NM_001127257	TC0200010328.hg.1
CALML4	0.30009294	NM_001031733	TC1500010886.hg.1
ZMIZ1	0.22366098	NM_020338	TC1000008182.hg.1
RWDD2A	0.24993981	NM_033411	TC0600008665.hg.1
ACLY	0.3235301	NM_001096	TC1700010693.hg.1
CENPF	0.15672778	NM_016343	TC0100011581.hg.1
PBK	0.3631735	NM_001278945	TC0800009970.hg.1
FECH	0.3086865	NM_000140	TC1800008776.hg.1
TMPRSS2	0.21594428	NM_001135099	TC2100008568.hg.1
RASSF3	0.21108563	NM_178169	TC1200008005.hg.1
TJP1	0.32519495	NM_001301026	TC1500008840.hg.1
STRN3	0.4758815	NM_001083893	TC1400008838.hg.1
topoy	0.13980052	topoy.aAug10-unspliced	TC1500010597.hg.1
CPNE3	0.29288474	NM_003909	TC0800008150.hg.1
EIF2S1	0.36441037	NM_004094	TC1400007494.hg.1
MKI67	0.16814242	NM_001145966	TC1000012235.hg.1
ACOT2	0.25482523	NM_006821	TC1400007647.hg.1
gyby	0.17672156	gyby.aAug10-unspliced	TC0Y00007075.hg.1
RSL24D1	0.45835865	NM_016304	TC1500009492.hg.1
CENPN	0.3301477	NM_001100624	TC1600008567.hg.1
CANX	0.25026622	NM_001024649	TC0500009700.hg.1
PRUNE2	0.17583582	NM_001308047	TC0900010470.hg.1
FLVCR1	0.38105702	NM_014053	TC0100011549.hg.1
SNORD108	0.31748018	NR_001292	TC1500006569.hg.1
PTTG1	0.27127957	NM_001282382	TC0500009281.hg.1
gawflubu	0.26713425	gawflubu.aAug10-unspliced	TC1200008010.hg.1
NOP56	0.43659714	NM_006392	TC2000006518.hg.1
RAB27A	0.34300104	NM_004580	TC1500010866.hg.1
SORD2P	0.2979169	ENST00000558556	TC1500009271.hg.1
CREB3L4	0.21225657	NM_001255978	TC0100010058.hg.1
PRR11	0.31903467	NM_018304	TC1700012285.hg.1
skawvybu	0.34753427	skawvybu.aAug10-unspliced	TC1700009030.hg.1
IL1R1	0.29786736	NM_000877	TC0200008666.hg.1
BUD13P1	0.327071	ENST00000603676	TC1100008833.hg.1
PTGFR	0.21314067	NM_000959	TC0100008812.hg.1
weysperby	0.18594058	weysperby.aAug10-unspliced	TC0400006510.hg.1
CORO1B	0.3642342	NM_001018070	TC1100013194.hg.1
ENOX1	0.4679353	NM_001127615	TC1300008766.hg.1
BDP1	0.23886979	NM_018429	TC0500007725.hg.1

Gene Symbol	Log2 ratio C42B-R vs. C42B Parental	mRNA Accession	Probe Set ID
GREB1	0.16320696	NM_014668	TC0200006725.hg.1
ASPM	0.21743447	NM_001206846	TC0100016831.hg.1
SPRY1	0.2334271	NM_001258038	TC0400008628.hg.1
snasney	0.25396127	snasney.aAug10-unspliced	TC1600011281.hg.1
ATP8A2	0.1917579	NM_016529	TC1300006643.hg.1
RAB3B	0.35519183	NM_002867	TC0100014191.hg.1
SNRPGP14	0.41755953	ENST00000435400	TC1300007166.hg.1
chysloby	0.2265794	chysloby.aAug10-unspliced	TC0500011649.hg.1
zarta	0.29425803	zarta.aAug10-unspliced	TC2100007211.hg.1
CAMKK2	0.1714699	NM_001270485	TC1200012199.hg.1
PARP1	0.35054034	NM_001618	TC0100017528.hg.1
snervobo	0.17004317	snervobo.aAug10-unspliced	TC0400008186.hg.1
ATAD2	0.22080632	NM_014109	TC0800011679.hg.1
weyta	0.26925874	weyta.aAug10-unspliced	TC2100008208.hg.1
SMS	0.2649469	NM_001258423	TC0X00006770.hg.1
MALT1	0.36564657	NM_006785	TC1800007431.hg.1
SLC44A4	0.19461238	ENST00000229729	TC0600011464.hg.1
GLUD1P3	0.35461098	NR_048575	TC1000008044.hg.1
jorsubu	0.3946707	jorsubu.aAug10-unspliced	TC2000007999.hg.1
FAM8A1	0.3006653	NM_016255	TC0600007092.hg.1
BCL6	0.36069652	NM_001130845	TC0300013471.hg.1
HOXB13	0.35708195	NM_006361	TC1700011004.hg.1
NPEPPS	0.37399438	NM_006310	TC1700008128.hg.1
PDE10A	0.36599797	NM_001130690	TC0600014366.hg.1
HIST1H1B	0.24172328	NM_005322	TC0600011232.hg.1
ADAMTS1	0.29155067	NM_006988	TC2100007821.hg.1
UTRN	0.2289929	NM_007124	TC0600009712.hg.1
lorby	0.17849019	lorby.aAug10-unspliced	TC0Y00007076.hg.1
TXLNGY	0.24385932	NR_045128	TC0Y00006722.hg.1
THBS1	0.29709524	NM_003246	TC1500006925.hg.1
KLK2	0.36345062	NM_001002231	TC1900008608.hg.1
TXNRD2	0.2685855	NM_001282512	TC2200008001.hg.1
sharbo	0.2386192	sharbo.aAug10-unspliced	TC0Y00007303.hg.1
blerbo	0.2386192	blerbo.aAug10-unspliced	TC0Y00007308.hg.1
PNKD	0.29503325	NM_001077399	TC0200010791.hg.1
teypoy	0.36982593	teypoy.aAug10-unspliced	TC1500010601.hg.1
TIMM21	0.47899324	NM_014177	TC1800007667.hg.1
ECI2	0.34800848	NM_001166010	TC0600010647.hg.1
KRR1	0.40118256	NM_007043	TC1200011301.hg.1
ATP5A1	0.35740972	NM_001001935	TC1800008584.hg.1
ERGIC1	0.35517403	NM_001031711	TC0500009470.hg.1
TMEM144	0.36965886	NM_018342	TC0400012854.hg.1
SERINC5	0.23867491	NM_001174071	TC0500011260.hg.1
SLC25A6	0.46236864	NM_001636	TC0Y00006882.hg.1
DSC2	0.2763578	NM_004949	TC1800009268.hg.1
RPS16P5	0.41788086	NR_046241	TC0600012073.hg.1
CKMT1A	0.38017067	NM_001015001	TC1500007067.hg.1
ARF6	0.324121	NM_001663	TC1400007098.hg.1
juber	0.49661344	juber.aAug10-unspliced	TC0100006832.hg.1
H2AFY2	0.44288647	NM_018649	TC1000007913.hg.1
PIGH	0.4095004	NM_004569	TC1400009490.hg.1
MCCC2	0.3196483	NM_022132	TC0500007729.hg.1
CDC20	0.28666273	NM_001255	TC0100008057.hg.1
ZBTB24	0.26842353	NM_001164313	TC0600014332.hg.1

Gene Symbol	Log2 ratio C42B-R vs. C42B Parental	mRNA Accession	Probe Set ID
SIM2	0.5361881	NM_005069	TC2100007056.hg.1
NKX3-1	0.16435982	NM_001256339	TC0800009885.hg.1
XRCC3	0.27602476	NM_001100118	TC1400010331.hg.1
BUB1	0.37584952	NM_001278616	TC0200013878.hg.1
SLC10A7	0.449806	NM_001029998	TC0400012057.hg.1
timemi	0.38455427	timemi.aAug10-unspliced	TC1300008339.hg.1
PRKD1	0.42288196	NM_002742	TC1400008816.hg.1
GPC6	0.29989374	NM_005708	TC1300007718.hg.1
RNU7-48P	0.4031717	uc063pkx.1	TC0600008470.hg.1
SNX4	0.50013524	NM_003794	TC0300012252.hg.1
LRIG1	0.17873994	NM_015541	TC0300011423.hg.1
ARHGAP11B	0.22825971	NM_001039841	TC1500006785.hg.1
munara	0.3707642	munara.aAug10-unspliced	TC2100007042.hg.1
SLC45A2	0.177171	NM_001297417	TC0500010456.hg.1
koysno	0.23974797	koysno.aAug10-unspliced	TC1600010318.hg.1
ANP32A	0.53166085	NM_006305	TC1500009865.hg.1
HMGB1	0.4518829	NM_002128	TC1300008517.hg.1
PFN1	0.41848212	NM_005022	TC1700009539.hg.1
NUCKS1	0.34123877	NM_022731	TC0100017075.hg.1
CD2AP	0.41604674	NM_012120	TC0600008183.hg.1
KIF20A	0.3513622	NM_005733	TC0500008777.hg.1
FGD4	0.3367115	NM_001304480	TC1200012617.hg.1
IL1RAPL1	0.20057039	NM_014271	TC0X00006865.hg.1
DIAPH3	0.34201103	NM_001042517	TC1300009088.hg.1
MIR4458	0.50332433	NR_039663	TC0500006676.hg.1
TDG	0.47353253	NM_003211	TC1200008669.hg.1
KIF15	0.33166784	ENST00000631233	TSUnmapped00000627.hg.1
MLPH	0.30814454	NM_001042467	TC0200011251.hg.1
NEDD4L	0.33894095	NM_001144964	TC1800007411.hg.1
plerkyby	0.3199108	plerkyby.aAug10-unspliced	TC1200008011.hg.1
CLN6	0.34336314	NM_017882	TC1500010887.hg.1
SLC25A33	0.48149735	NM_032315	TC0100006781.hg.1
SENP7	0.50948095	NM_001077203	TC0300011853.hg.1
YBX1	0.5049476	NM_004559	TC0100008025.hg.1
zarjyby	0.3353358	zarjyby.aAug10-unspliced	TC1200011358.hg.1
ABHD14A-ACY1	0.43702936	OTTHUMT00000349691	TC0300013840.hg.1
sputa	0.29172438	sputa.aAug10-unspliced	TC1500007807.hg.1
TYMS	0.2758343	NM_001071	TC1800006448.hg.1
WWC1	0.3649045	NM_001161661	TC0500009346.hg.1
NEK9	0.46871623	NM_033116	TC1400009711.hg.1
MELK	0.31517687	NM_001256685	TC0900007129.hg.1
MOAP1	0.43198103	NM_022151	TC1400010019.hg.1
RRM2	0.28806388	NM_001034	TC0200006677.hg.1
DAPK1	0.36082718	NM_001288729	TC0900007807.hg.1
rini	0.4190027	rini.aAug10-unspliced	TC0200008897.hg.1
ACSL3	0.3783435	NM_004457	TC0200010907.hg.1
NEK2	0.31032336	NM_001204182	TC0100017216.hg.1
EPHA3	0.27893412	NM_005233	TC0300008029.hg.1
ILF2	0.5002116	NM_001267809	TC0100015875.hg.1
IFT88	0.53109074	NM_006531	TC1300006503.hg.1
ESRP2	0.36685258	NM_024939	TC1600010676.hg.1
NTNG1	0.21399915	NM_001113226	TC0100009307.hg.1
DEFB134	0.49540353	NM_001302695	TC0800009626.hg.1
FBXL17	0.43809125	NM_001163315	TC0500011655.hg.1

Gene Symbol	Log2 ratio C42B-R vs. C42B Parental	mRNA Accession	Probe Set ID
mimira	0.48947382	mimira.aAug10-unspliced	TC0300012539.hg.1
KIF23	0.37375492	NM_001281301	TC1500007699.hg.1
TAOK3	0.13655189	NM_016281	TC1200012111.hg.1
ANLN	0.2862607	NM_001284301	TC0700007198.hg.1
SEC23A	0.45338348	NM_006364	TC1400009002.hg.1
DDX24	0.4291318	NM_020414	TC1400010039.hg.1
foytaby	0.40464175	foytaby.aAug10-unspliced	TC0900011475.hg.1
PTCH1	0.35308182	NM_000264	TC0900010886.hg.1
CCNB1	0.25894988	NM_031966	TC0500007665.hg.1
roydy	0.49512893	roydy.aAug10-unspliced	TC0100011339.hg.1
MTCO3P2	0.3840261	ENST00000457365	TC1300008341.hg.1
ELF5	0.40135887	NM_001243080	TC1100010514.hg.1
BEND4	0.31355995	NM_001159547	TC0400010553.hg.1
KLK3	0.28711143	NM_001030047	TC1900008607.hg.1
HIST1H2AG	0.22220252	NM_021064	TC0600014083.hg.1
VPS13A	0.34589922	NM_001018037	TC0900007646.hg.1
KIAA0391	0.5122039	NM_001256678	TC1400010595.hg.1
SNORD116-19	0.34560978	NR_001290	TC1500010700.hg.1
CDK8	0.38814873	NM_001260	TC1300006652.hg.1
MTMR6	0.3956666	NM_004685	TC1300008386.hg.1
sukey	0.4230447	sukey.aAug10-unspliced	TC2000009724.hg.1
SGK3	0.25628427	NM_001033578	TC0800012313.hg.1
SFPQ	0.43931386	NM_005066	TC0100018432.hg.1
BMPR1B	0.23123476	NM_001203	TC0400008183.hg.1
ZNF24	0.42688167	NM_001308123	TC1800008458.hg.1
LRPPRC	0.5606997	NM_133259	TC0200012418.hg.1
IARS2	0.45644376	NM_018060	TC0100011648.hg.1
FAM72C	0.3270693	NM_001287385	TC0100015550.hg.1
CCDC59	0.4835724	NM_014167	TC1200011400.hg.1
HEBP2	0.375916	NM_014320	TC0600009611.hg.1
SAP18	0.5344174	NM_005870	TC1300006520.hg.1
SORD	0.3865239	NM_003104	TC1500007107.hg.1
CTD-2165H16.3	0.3164789	ENST00000503819	TC0500006818.hg.1
RACGAP1	0.32185745	NM_001126103	TC1200010650.hg.1
narrobo	0.49617535	narrobo.aAug10	TC0100007056.hg.1
CLPX	0.45414108	NM_006660	TC1500009785.hg.1
HJURP	0.3543657	NM_001282962	TC0200016099.hg.1
smoydoybu	0.29765475	smoydoybu.aAug10-unspliced	TC0200016199.hg.1
RDH11	0.28973332	NM_001252650	TC1400010765.hg.1
FOXA1	0.33198506	NM_004496	TC1400008981.hg.1
DENND1A	0.48897478	NM_020946	TC0900011473.hg.1
foyboyby	0.46238762	foyboyby.aAug10-unspliced	TC1400009947.hg.1
RAN	0.6184955	NM_001300796	TC1200009416.hg.1
LIFR	0.29280463	NM_001127671	TC0500010540.hg.1
natobo	0.44247767	natobo.aAug10-unspliced	TC1100009113.hg.1
teesparbu	0.5659554	teesparbu.aAug10-unspliced	TC0600009212.hg.1
ZNF271P	0.5083222	NR_024565	TC1800009224.hg.1
RP11-299A16.1	0.39356023	ENST00000549144	TC1200011387.hg.1
MAPK12	0.5344366	NM_001303252	TC2200009361.hg.1
NR3C2	0.27024943	NM_000901	TC0400012078.hg.1
ANK3	0.4311011	NM_001149	TC1000010727.hg.1
ABCC1	0.34423995	ENST00000399408	TC1600007022.hg.1
AKT1	0.42122245	NM_001014431	TC1400010377.hg.1
GUCY1A3	0.3828525	NM_000856	TC0400009086.hg.1

Gene Symbol	Log2 ratio C42B-R vs. C42B Parental	mRNA Accession	Probe Set ID
SKA2	0.49627495	NM_001100595	TC1700011281.hg.1
PHF8	0.37629598	NM_001184896	TC0X00009773.hg.1
SLC41A1	0.35808146	NM_173854	TC0100017079.hg.1
fleejybu	0.3452867	fleejybu.aAug10-unspliced	TC0200013632.hg.1
gospoy	0.32074985	gospoy.aAug10-unspliced	TC1500007028.hg.1
NFIB	0.3704507	NM_001190737	TC0900009569.hg.1
CNKSR2	0.3673903	NM_001168647	TC0X00006758.hg.1
jeezaw	0.421056	jeezaw.aAug10-unspliced	TC1500006568.hg.1
ETFB	0.41879007	NM_001014763	TC1900012016.hg.1
ZNF397	0.48176062	NM_001135178	TC1800007071.hg.1
RP11-134K13.2	0.38016275	ENST00000454078	TC0600012254.hg.1
HNRNPAB	0.34353036	NM_004499	TC0500009652.hg.1
pynawbu	0.44925985	pynawbu.aAug10-unspliced	TC0100006885.hg.1
KPNB1	0.33728483	NM_001276453	TC1700008133.hg.1
TPT1P2	0.5830669	ENST00000413105	TC0200015576.hg.1
ZC2HC1C	0.59368634	NM_001042430	TC1400007698.hg.1
BAG1	0.45525038	NM_001172415	TC0900009855.hg.1
noynoy	0.4063627	noynoy.aAug10-unspliced	TC1300009675.hg.1
SNRPA1	0.5292234	NM_003090	TC1500010659.hg.1
FANCD2	0.45731786	NM_001018115	TC0300006575.hg.1
TBRG1	0.58383816	NM_032811	TC1100009395.hg.1
UTY	0.3506408	NM_001258249	TC0Y00007074.hg.1
CYC1	0.40678605	NM_001916	TC0800009239.hg.1
GABBR1	0.57983565	NM_021903	TC0600014256.hg.1
PALMD	0.4039666	NM_017734	TC0100009183.hg.1
CXCL12	0.28160012	NM_000609	TC1000010468.hg.1
RNF157	0.5439441	NM_052916	TC1700011787.hg.1
FUZ	0.37984273	NM_001171937	TC1900012001.hg.1
ANGEL2	0.41840667	NM_001300753	TC0100017267.hg.1
PRKCD	0.39211175	NM_006254	TC0300007512.hg.1
KLHL20	0.39303213	NM_014458	TC0100010654.hg.1
KIF22	0.3722794	NM_001256269	TC1600007425.hg.1
NXPE3	0.35135555	NM_001134456	TC0300013854.hg.1
reymeybo	0.56688315	reymeybo.aAug10-unspliced	TC0100012420.hg.1
TCP1	0.40203378	NM_001008897	TC0600013762.hg.1
RMDN1	0.42244607	NM_001286707	TC0800011021.hg.1
NDUFB10	0.33624062	NM_004548	TC1600006574.hg.1
EMC9	0.47888026	NM_016049	TC1400010720.hg.1
NUF2	0.26165643	NM_031423	TC0100010397.hg.1
RAB4A	0.36328965	NM_001271998	TC0100011901.hg.1
NGDN	0.47207433	NM_001042635	TC1400010577.hg.1
AC254944.3	0.25866434	ENST00000633373	TSUnmapped00000642.hg.1
MICAL2	0.37170386	NM_001282663	TC1100012966.hg.1
APEX1	0.35886616	NM_001244249	TC1400006519.hg.1
TMEM106C	0.5600749	NM_001143841	TC1200007499.hg.1
SNX5	0.43134022	NM_001282454	TC2000009986.hg.1
EPPK1	0.36392793	NM_031308	TC0800012173.hg.1
ANKRD29	0.45737383	NM_001308238	TC1800008287.hg.1
larklabu	0.45057094	larklabu.aAug10-unspliced	TC1000008187.hg.1
KLF5	0.32394165	NM_001286818	TC1300007491.hg.1
PLK4	0.48964557	NM_001190799	TC0400008664.hg.1
TPD52	0.5001518	NM_001025252	TC0800012433.hg.1
PEBP1	0.41023007	NM_002567	TC1200009071.hg.1
GLUD1	0.41531926	NM_005271	TC1000011323.hg.1

Gene Symbol	Log2 ratio C42B-R vs. C42B Parental	mRNA Accession	Probe Set ID
ZFYVE21	0.3636023	NM_001198953	TC1400008415.hg.1
RIOK3	0.45406142	NM_003831	TC1800006889.hg.1
TMEM141	0.5360678	NM_032928	TC0900012189.hg.1
ECT2	0.38334417	NM_001258315	TC0300009524.hg.1
ADK	0.62799495	NM_001123	TC1000008063.hg.1
PRKAR1B	0.35480964	NM_001164758	TC0700009980.hg.1
CNTN3	0.3065296	NM_020872	TC0300011566.hg.1
GSS	0.5023408	NM_000178	TC2000008951.hg.1
hetura	0.42098624	hetura.aAug10-unspliced	TC0900010567.hg.1
steesawby	0.3039175	steesawby.aAug10-unspliced	TC0600007620.hg.1
ARHGEF26	0.43793955	NM_001251962	TC0300009247.hg.1
PDE9A	0.33857653	NM_001001567	TC2100007263.hg.1
yaka	0.40206742	yaka.aAug10-unspliced	TC0400012080.hg.1
ADAM9	0.5030958	NM_003816	TC0800007367.hg.1
EFCAB12	0.238082	NM_207307	TC0300012392.hg.1
TLK1	0.3972267	NM_001136554	TC0200014893.hg.1
MTHFD1	0.46834892	NM_005956	TC1400007440.hg.1
UBE2T	0.43260032	NM_001310326	TC0100016952.hg.1
SLC39A6	0.38609526	NM_001099406	TC1800008474.hg.1
ELOVL5	0.49991712	NM_001242828	TC0600012072.hg.1
ZNF217	0.4334751	NM_006526	TC2000009504.hg.1
PAK1IP1	0.46362332	NM_017906	TC0600006926.hg.1
RUVBL1	0.53666615	NM_003707	TC0300014041.hg.1
POLQ	0.3772788	NM_199420	TC0300012164.hg.1
RAD51	0.46302542	NM_001164269	TC1500006965.hg.1
CKAP2	0.30626553	NM_001098525	TC1300007248.hg.1
FAM21C	0.59869045	NM_001169106	TC1000007483.hg.1
flasmee	0.55927515	flasmee.aAug10-unspliced	TC1700007664.hg.1
CRNKL1	0.45674998	NM_001278625	TC2000008573.hg.1
ACADSB	0.41667643	NM_001609	TC1000009169.hg.1
PIAS2	0.5429803	NM_004671	TC1800009277.hg.1
spofeybu	0.3030643	spofeybu.aAug10-unspliced	TC0200010153.hg.1
TMEM87A	0.4031513	NM_001110503	TC1500009185.hg.1
STMN1	0.4238215	NM_001145454	TC0100013369.hg.1
ISYNA1	0.44977745	NM_001170938	TC1900010016.hg.1
RIPK4	0.2818225	NM_020639	TC2100008222.hg.1
FAM111A	0.4508963	NM_001142519	TC1100007728.hg.1
ZFYVE16	0.4000161	NM_001105251	TC0500007912.hg.1
MYO6	0.41921926	NM_001300899	TC0600008569.hg.1
PPP4R1	0.47668493	NM_001042388	TC1800008035.hg.1
C1QTNF3	0.28285766	NM_030945	TC0500013298.hg.1
EZR	0.36998093	NM_001111077	TC0600013727.hg.1
DCXR	0.2795626	NM_001195218	TC1700012089.hg.1
DDT	0.3362822	NM_001084392	TC2200008203.hg.1
TTC21B	0.3856894	NM_024753	TC0200016747.hg.1
MCCC1	0.513701	NM_001293273	TC0300013334.hg.1
MIPOL1	0.4788423	ENST00000624539	TC1400006945.hg.1
FAM103A1	0.47701406	BC003627_2	TC0600013898.hg.1
ZBTB16	0.3234695	NM_001018011	TC1100009101.hg.1
SBNO1	0.5172215	NM_001167856	TC1200012267.hg.1
SLC24A5	0.48911297	NM_205850	TC1500007156.hg.1
titari	0.3180082	titari.aAug10-unspliced	TC1700010782.hg.1
suraw	0.5245537	suraw.aAug10-unspliced	TC2000007871.hg.1
BAZ1A	0.5356686	NM_013448	TC1400008921.hg.1

Gene Symbol	Log2 ratio C42B-R vs. C42B Parental	mRNA Accession	Probe Set ID
KIF15	0.3740754	NM_020242	TC0300007223.hg.1
SLC4A4	0.29856068	NM_001098484	TC0400007799.hg.1
GRK1	0.55618113	BC140945	TC1300008149.hg.1
SPDL1	0.43520954	NM_017785	TC0500009377.hg.1
ADSL	0.41026914	NM_000026	TC2200009275.hg.1
skarsteyby	0.443283	skarsteyby.aAug10-unspliced	TC0500008264.hg.1
ALDH6A1	0.36875987	NM_001278593	TC1400009684.hg.1
EVL	0.5096684	NM_016337	TC1400008193.hg.1
TTC6	0.34719765	NM_001310135	TC1400010599.hg.1
SLC25A6	0.49791104	ENST00000381401	TC0X00008908.hg.1
VASH2	0.4435645	NM_001136474	TC0100011552.hg.1
PRMT5	0.48022914	NM_001039619	TC1400008684.hg.1
NDUFV1	0.39669672	NM_001166102	TC1100008147.hg.1
skaspeyby	0.30640018	skaspeyby.aAug10-unspliced	TC0600008864.hg.1
swerslyby	0.3140467	swerslyby.aAug10-unspliced	TC0300011837.hg.1
LARP7	0.5211242	NM_001267039	TC0400008443.hg.1
leebly	0.5281036	leebly.aAug10-unspliced	TC1800009205.hg.1
HEATR5A	0.45703176	NM_015473	TC1400010728.hg.1
PIK3AP1	0.37018564	NM_152309	TC1000011524.hg.1
sleyblaby	0.30314198	sleyblaby.aAug10-unspliced	TC0500011736.hg.1
HMGN2	0.51322186	NM_005517	TC0100007463.hg.1
PPM1A	0.61204994	NM_021003	TC1400007354.hg.1
KIAA0101	0.31054652	NM_001029989	TC1500009738.hg.1
CAPS2	0.5079297	NM_001286547	TC1200011298.hg.1
AURKA	0.32011145	NM_003600	TC2000009550.hg.1
skoter	0.55102247	skoter.aAug10-unspliced	TC1500008777.hg.1
RPS6KC1	0.36114416	NM_001136138	TC0100011554.hg.1
NCAPD3	0.62688273	NM_015261	TC1100012917.hg.1
spawsmoybu	0.21681145	spawsmoybu.aAug10-unspliced	TC0700008883.hg.1
PSMA4	0.46559218	NM_001102667	TC1500007979.hg.1
IVD	0.36071888	NM_001159508	TC1500010720.hg.1
UQCRC1	0.4459037	NM_003365	TC0300011029.hg.1
HIST1H3I	0.30473334	NM_003533	TC0600011233.hg.1
skoyklee	0.4531795	skoyklee.aAug10-unspliced	TC1500009624.hg.1
ANKRD18B	0.5432424	NM_001244752	TC0900006986.hg.1
woysperby	0.34512398	woysperby.aAug10-unspliced	TC0400009724.hg.1
FAM72D	0.31749475	NM_207418	TC0100009723.hg.1
HMGN2P22	0.5714515	ENST00000427588	TC0200013645.hg.1
ACY1	0.37561074	NM_000666	TC0300013839.hg.1
EFCAB11	0.46855548	NM_001284266	TC1400010775.hg.1
RAD23B	0.49676886	NM_001244713	TC0900008345.hg.1
HIST1H4C	0.35986647	NM_003542	TC0600007268.hg.1
PA2G4	0.49768442	NM_006191	TC1200012644.hg.1
CTD-2089O24.2	0.5559397	ENST00000509899	TC0500011693.hg.1
DIP2A	0.5080272	NM_001146115	TC2100007467.hg.1
GALK2	0.4717398	NM_001001556	TC1500007180.hg.1
GFM1	0.5042876	NM_001308164	TC0300009313.hg.1
ECHDC3	0.4734487	NM_024693	TC1000006782.hg.1
BIRC5	0.45699316	NM_001012270	TC1700009043.hg.1
SDR39U1	0.48289615	NM_001290292	TC1400008760.hg.1
AS3MT	0.5401746	NM_020682	TC1000012496.hg.1
LOC440434	0.52549684	NR_036750_3	TC1700010516.hg.1
MPHOSPH9	0.35958567	NM_022782	TC1200012263.hg.1
HMGN2P6	0.48975417	OTTHUMT00000408473	TC1400008772.hg.1

Gene Symbol	Log2 ratio C42B-R vs. C42B Parental	mRNA Accession	Probe Set ID
KIF20B	0.42841735	NM_001284259	TC1000008406.hg.1
GTF2I	0.55467445	NM_001163636	TC0700008010.hg.1
TBC1D8	0.44864577	NM_001102426	TC0200016717.hg.1
DCAF6	0.4392419	NM_001017977	TC0100010518.hg.1
swypoby	0.3998323	swypoby.aAug10-unspliced	TC1400007502.hg.1
ACIN1	0.53526825	NM_001164814	TC1400008694.hg.1
GCFC2	0.41635793	NM_001201334	TC0200013137.hg.1
GET4	0.35419556	NM_015949	TC0700013324.hg.1
NCAPG	0.36457863	NM_022346	TC0400006994.hg.1
FPGS	0.40481937	NM_001018078	TC0900008827.hg.1
CIPC	0.487878	NM_033426	TC1400010634.hg.1
FAM72B	0.32704642	NM_001100910	TC0100015509.hg.1
morzorby	0.4562262	morzorby.aAug10-unspliced	TC0500007642.hg.1
UBE2E4P	0.39943308	NR_110506	TC0X00006666.hg.1
WLS	0.47495472	NM_001002292	TC0100014531.hg.1
CDH26	0.44236112	NM_021810	TC2000007943.hg.1
narna	0.41452712	narna.aAug10-unspliced	TC1100012367.hg.1
swosa	0.517973	swosa.aAug10	TC1700011559.hg.1
kawwer	0.570126	kawwer.aAug10-unspliced	TC1700010483.hg.1
flarabu	0.3971643	flarabu.aAug10-unspliced	TC0100017071.hg.1
ACACA	0.4545591	NM_198834	TC1700010488.hg.1
moromi	0.35541314	moromi.aAug10-unspliced	TC1400008821.hg.1
RP11-996F15.4	0.4840695	OTTHUMT00000474523	TC1200010246.hg.1
HIST2H3A	0.30260772	ENST00000403683	TC0100009877.hg.1
glorgler	0.5126461	glorgler.aAug10-unspliced	TC1100009830.hg.1
PMCHL2	0.30508903	NR_003922	TC0500007722.hg.1
florgar	0.58892053	florgar.aAug10-unspliced	TC0X00010585.hg.1
bygar	0.58892053	bygar.aAug10-unspliced	TC0X00010578.hg.1
jubabu	0.3765409	jubabu.aAug10-unspliced	TC0300008185.hg.1
DNAH5	0.23655511	NM_001369	TC0500010148.hg.1
C1QBP	0.58807564	NM_001212	TC1700012341.hg.1
CENPJ	0.4520132	NM_018451	TC1300008371.hg.1
NR6A1	0.54203063	NM_001278546	TC0900011501.hg.1
RP11-490K7.1	0.5037431	ENST00000441274	TC0100013554.hg.1
smuswawbu	0.41096988	smuswawbu.aAug10-unspliced	TC0500008713.hg.1
volo	0.31745464	volo.aAug10-unspliced	TC1800007419.hg.1
RBM12	0.47325093	NM_001198838	TC2000010007.hg.1
METTL3	0.45550555	NM_019852	TC1400008636.hg.1
TPX2	0.3373943	NM_012112	TC2000007089.hg.1
HGH1	0.44923848	NM_016458	TC0800009242.hg.1
CDC73	0.60068417	NM_024529	TC0100011027.hg.1
KPNA2	0.46684107	NM_002266	TC1700008719.hg.1
reeteebo	0.43674248	reeteebo.aAug10-unspliced	TC0900011112.hg.1
cheeklar	0.5044209	cheeklar.aAug10-unspliced	TC1800007906.hg.1
rerplar	0.37952906	rerplar.aAug10-unspliced	TC1100007871.hg.1
HN1	0.45646855	NM_001002032	TC1700012468.hg.1
RP11-380B4.3	0.33276784	OTTHUMT00000474773	TC1300006813.hg.1
FAM216A	0.5550853	NM_013300	TC1200008843.hg.1
swawchu	0.33273834	swawchu.aAug10-unspliced	TC1200009726.hg.1
ACOT1	0.44290358	NM_001037161	TC1400007644.hg.1
ARFGEF3	0.38207057	NM_020340	TC0600009606.hg.1
SLC2A3P1	0.589518	ENST00000519666	TC0500013398.hg.1
LRRC58	0.4514726	NM_001099678	TC0300012143.hg.1
C15orf61	0.5366675	NM_001143936	TC1500007644.hg.1

Gene Symbol	Log2 ratio C42B-R vs. C42B Parental	mRNA Accession	Probe Set ID
COPS3	0.5833752	NM_001199125	TC1700009928.hg.1
ATP6V1G2-DDX39B	0.47000057	OTTHUMT00000355085	TC0600014259.hg.1
SLC25A30	0.35315812	NM_001010875	TC1300008824.hg.1
PLCB4	0.3078938	NM_000933	TC2000006674.hg.1
SIRT1	0.48724964	NM_001142498	TC1000007846.hg.1
ferborbo	0.5128306	ferborbo.aAug10-unspliced	TC0400012474.hg.1
vawslorbu	0.40539122	vawslorbu.aAug10-unspliced	TC0700010158.hg.1
HIST2H3A	0.28990564	NM_001005464	TC0100015701.hg.1
doyskabu	0.3917938	doyskabu.aAug10-unspliced	TC0900012071.hg.1
SNW1	0.5053038	NM_012245	TC1400009793.hg.1
SRGAP1	0.54046434	NM_020762	TC1200007992.hg.1
RCN2	0.58248293	NM_001271837	TC1500007926.hg.1
snogarbu	0.39408144	snogarbu.aAug10-unspliced	TC0200015740.hg.1
CBS	0.49238095	NM_001178008	TC2100008285.hg.1
USP22	0.5444251	NM_015276	TC1700010076.hg.1
NSMAF	0.5124762	NM_001144772	TC0800010553.hg.1
snamu	0.50297415	snamu.aAug10-unspliced	TC1000008093.hg.1
CTPS2	0.5248332	NM_001144002	TC0X00009153.hg.1
THOC3	0.442024	NM_032361	TC0500013414.hg.1
CPNE7	0.3142505	NM_014427	TC1600008889.hg.1
shuferbu	0.55468684	shuferbu.aAug10-unspliced	TC0300012858.hg.1
chawshaby	0.383499	chawshaby.aAug10-unspliced	TC0500009473.hg.1
THOC5	0.6010576	NM_001002877	TC2200009335.hg.1
RP11-492D6.3	0.50888956	ENST00000557586	TC1500008611.hg.1
SNRPN	0.5263334	NM_003097	TC1500010698.hg.1
FAM103A1	0.46384513	NM_031452	TC1500008105.hg.1
UNC5A	0.49364874	NM_133369	TC0500009603.hg.1
FAM50B	0.60161567	NM_012135	TC0600006714.hg.1
SFXN2	0.38283017	NM_178858	TC1000008749.hg.1
HMG20B	0.3193636	NM_006339	TC1900006645.hg.1
CDC42SE1	0.5644814	NM_001038707	TC0100018486.hg.1
FAM179B	0.40688616	NM_001308120	TC1400007033.hg.1
DHFR	0.5037779	BC070280	TC1800008333.hg.1
IGF1	0.388195	NM_000618	TC1200011727.hg.1
basko	0.546372	basko.aAug10-unspliced	TC1800009037.hg.1
PPP5C	0.4715483	NM_001204284	TC1900008372.hg.1
RFX7	0.4799321	NM_022841	TC1500009514.hg.1
GNG5	0.64146954	NM_005274	TC0100014752.hg.1
HIST1H4J	0.4420709	NM_021968	TC0600007378.hg.1
RP11-292K15.1	0.4901269	ENST00000604494	TC0200008611.hg.1
CCDC88C	0.42444608	NM_001080414	TC1400009980.hg.1
HELLS	0.4641056	NM_001289067	TC1000012477.hg.1
ZFY	0.41554436	NM_001145275	TC0Y00006487.hg.1
ZNHIT3	0.6281227	NM_001281432	TC1700007634.hg.1
NVL	0.3446228	NM_001243146	TC0100017467.hg.1
LPAR3	0.43545845	NM_012152	TC0100014766.hg.1
NPPC	0.44196656	NM_024409	TC0200016047.hg.1
vowaw	0.44001612	vowaw.aAug10-unspliced	TC2000008363.hg.1
AP4S1	0.53365564	NM_001128126	TC1400006833.hg.1
ZNF850	0.37326476	NM_001193552	TC1900010533.hg.1
USP14	0.50795335	NM_001037334	TC1800006437.hg.1
jawwu	0.40663308	jawwu.aAug10-unspliced	TC1800007865.hg.1
snoydarbu	0.41579497	snoydarbu.aAug10-unspliced	TC0200012965.hg.1
MRRF	0.58635247	NM_001173512	TC0900012168.hg.1

Gene Symbol	Log2 ratio C42B-R vs. C42B Parental	mRNA Accession	Probe Set ID
PEBP1P2	0.45017985	OTTHUMT00000468819	TC0200013260.hg.1
GMNN	0.51811886	NM_001251989	TC0600007207.hg.1
bypla	0.49483213	bypla.aAug10-unspliced	TC1800006709.hg.1
RP11-511H9.3	0.4667998	ENST00000552036	TC1200011713.hg.1
STK39	0.45183507	NM_013233	TC0200014834.hg.1
EIF2AK4	0.5377841	NM_001013703	TC1500006930.hg.1
marreybu	0.3624884	marreybu.aAug10-unspliced	TC0300011558.hg.1
DDTL	0.3729007	NM_001084393	TC2200006874.hg.1
ARHGAP11B	0.331973	BC105788	TC1500010707.hg.1
spuskerbu	0.44177732	spuskerbu.aAug10-unspliced	TC0800011666.hg.1
TBC1D3L	0.4612347	OTTHUMT00000477403	TC1700012395.hg.1
flokubo	0.40294802	flokubo.aAug10-unspliced	TC0200013843.hg.1
REPS2	0.355074	NM_001080975	TC0X00006704.hg.1
HIVEP1	0.44757175	NM_002114	TC0600014074.hg.1
FANCA	0.47377378	NM_000135	TC1600011292.hg.1
ITPK1	0.49310368	NM_001142593	TC1400010015.hg.1
N6AMT2	0.44141588	NM_174928	TC1300008264.hg.1
GINS1	0.3616711	NM_021067	TC2000007016.hg.1
RP11-49K24.5	0.5703032	ENST00000588843	TC1800007242.hg.1
ZWINT	0.4332938	NM_001005413	TC1000010686.hg.1
PDZRN3	0.37808138	NM_001303139	TC0300011550.hg.1
MRPL37	0.55341005	NM_016491	TC0100008383.hg.1
CDCA2	0.38652524	NM_152562	TC0800007065.hg.1
C1orf116	0.26104504	NM_001083924	TC0100017116.hg.1
PHF10	0.5350194	NM_018288	TC0600014374.hg.1
ANTXR1	0.41467232	NM_018153	TC0200007908.hg.1
GANC	0.4332393	NM_001301409	TC1500010724.hg.1
buney	0.21448067	buney.aAug10-unspliced	TC1200011096.hg.1
fleecha	0.5670341	fleecha.aAug10-unspliced	TC2200007375.hg.1
TOPBP1	0.5231038	NM_007027	TC0300012475.hg.1
DDHD1	0.518078	NM_001160147	TC1400009198.hg.1
CENPE	0.36218575	NM_001286734	TC0400011477.hg.1
steyswabu	0.39305443	steyswabu.aAug10-unspliced	TC0400012058.hg.1
LRRC41	0.5029992	NM_006369	TC0100014050.hg.1
chateebo	0.46080166	chateebo.aAug10-unspliced	TC0900011504.hg.1
DHCR24	0.29359323	NM_014762	TC0100014276.hg.1
C16orf47	0.61414534	NM_207385	TC1600010837.hg.1
ANKH	0.2953773	NM_054027	TC0500010169.hg.1
SPECC1L	0.4498078	NM_001145468	TC2200009232.hg.1
SURF2	0.58516955	ENST00000630633	TSUnmapped00000645.hg.1
ANKRD18A	0.447803	NM_147195	TC0900010056.hg.1
MRPL50P4	0.6036548	ENST00000446432	TC1000010721.hg.1
tawkoyby	0.46899974	tawkoyby.aAug10-unspliced	TC1500008742.hg.1
ploko	0.39340684	ploko.aAug10-unspliced	TC0700008118.hg.1
PGC	0.58806777	NM_001166424	TC0600011800.hg.1
FTL	0.49715453	NM_000146	TC1900008507.hg.1
C1QTNF9B-AS1	0.46001562	ENST00000626707	TC1300008354.hg.1
ANKRD16	0.4167979	NM_001009941	TC1000009685.hg.1
GRTP1	0.4795477	NM_001286732	TC1300009898.hg.1
DSG1-AS1	0.44299492	NR_110788	TC1800008391.hg.1
RABGGTA	0.49729913	NM_004581	TC1400008749.hg.1
flawkerbu	0.43726686	flawkerbu.aAug10-unspliced	TC0300009525.hg.1
HNRNPM	0.56403583	NM_001297418	TC1900006896.hg.1
SUPT16H	0.49480245	NM_007192	TC1400008628.hg.1

Gene Symbol	Log2 ratio C42B-R vs. C42B Parental	mRNA Accession	Probe Set ID
zorflo	0.3359878	zorflo.aAug10-unspliced	TC0500011265.hg.1
SLC43A1	0.38509548	NM_001198810	TC1100010893.hg.1
SMCHD1	0.51081747	NM_015295	TC1800006487.hg.1
AK5	0.30435124	NM_012093	TC0100008797.hg.1
ALYREF	0.56492716	NM_005782	TC1700012078.hg.1
galerby	0.45747203	galerby.aAug10-unspliced	TC1100009108.hg.1
wawplabu	0.42711604	wawplabu.aAug10-unspliced	TC0900011716.hg.1
PSMC1P5	0.5066039	ENST00000512850	TC0500008258.hg.1
RFX3	0.4212338	NM_001282116	TC0900009414.hg.1
PCYT2	0.47380823	NM_001184917	TC1700012079.hg.1
LIPT1	0.4635479	NM_001204830	TC0200016498.hg.1
PCNA	0.52286285	NM_002592	TC2000008279.hg.1
UAP1L1	0.47232428	NM_207309	TC0900009259.hg.1
ruyura	0.37359488	ruyura.aAug10-unspliced	TC0200011383.hg.1
TIMM9P1	0.45987964	ENST00000436122	TC1000011053.hg.1
POLR1D	0.5329166	NM_001206559	TC1300006690.hg.1
SPC25	0.38880345	NM_020675	TC0200014845.hg.1
SYNGR2	0.395677	NM_004710	TC1700009040.hg.1
GYS1	0.5489342	NM_001161587	TC1900011113.hg.1
FO082796.1	0.6236664	uc064xof.1	TC0900009337.hg.1
POLR3A	0.50877184	NM_007055	TC1000011135.hg.1
HMGCS2	0.40646768	NM_001166107	TC0100015476.hg.1
HMGB2	0.4243033	NM_001130688	TC0400012432.hg.1
SEC23IP	0.5455924	NM_007190	TC1000009095.hg.1
MND1	0.43333137	NM_001253861	TC0400009037.hg.1
skorby	0.5538103	skorby.aAug10-unspliced	TC0100008386.hg.1
MAPK6	0.58080816	NM_002748	TC1500010743.hg.1
RP11-523H24.3	0.40864602	ENST00000423023.2	TC1300006475.hg.1
NDUFS2	0.51578885	NM_001166159	TC0100010340.hg.1
THUMPD2	0.58832085	NM_025264	TC0200012335.hg.1
ZNF532	0.51026833	NM_018181	TC1800007440.hg.1
RNF31	0.4634247	NM_001310332	TC1400010583.hg.1
LMNB1	0.3586973	NM_001198557	TC0500008544.hg.1
NCL	0.43537042	NM_005381	TC0200016018.hg.1
C16orf70	0.4627001	NM_025187	TC1600008144.hg.1
ADPRHL1	0.49212754	NM_138430	TC1300009906.hg.1
DTL	0.5244777	NM_001286229	TC0100011512.hg.1
FBXO9	0.5130895	NM_012347	TC0600008272.hg.1
ARL4AP1	0.5907149	OTTHUMT00000048210	TC1000007746.hg.1
ARHGDIA	0.45357972	NM_001185077	TC1700012076.hg.1
CDCA8	0.40365022	NM_001256875	TC0100007841.hg.1
RP11-428P16.2	0.4777028	ENST00000394628	TC1000008141.hg.1
AFF3	0.48423874	NM_001025108	TC0200013616.hg.1
SNX6	0.6242106	NM_021249	TC1400008913.hg.1
SETDB2	0.46258482	NM_001160308	TC1300007164.hg.1
BCHE	0.37906632	NM_000055	TC0300013025.hg.1
SCARF2	0.4983522	NM_153334	TC2200009316.hg.1
bawwaw	0.5893574	bawwaw.aAug10-unspliced	TC1700009071.hg.1
duspoyby	0.4026641	duspoyby.aAug10-unspliced	TC0300007163.hg.1
CIRBP	0.4957005	NM_001280	TC1900006524.hg.1
leyreyby	0.57445896	leyreyby.aAug10-unspliced	TC1500010685.hg.1
TMEM38B	0.4955472	NM_018112	TC0900008318.hg.1
gupla	0.5598206	gupla.aAug10-unspliced	TC1500009774.hg.1
HIST2H2BF	0.47186053	NM_001024599	TC0100015696.hg.1

Gene Symbol	Log2 ratio C42B-R vs. C42B Parental	mRNA Accession	Probe Set ID
XPO6	0.55591255	NM_001270940	TC1600009849.hg.1
plerdoby	0.5506005	plerdoby.aAug10-unspliced	TC1300007746.hg.1
HIST1H2BJ	0.31735694	NM_021058	TC0600011184.hg.1
jorsno	0.3005757	jorsno.aAug10-unspliced	TC1600010317.hg.1
TK1	0.38798255	NM_003258	TC1700011892.hg.1
QARS	0.4150439	NM_001272073	TC0300011050.hg.1
DNAJA2	0.65016097	NM_005880	TC1600010179.hg.1
ZNF511	0.50889003	NM_145806	TC1000012510.hg.1
IDH3A	0.4949564	NM_005530	TC1500010776.hg.1
PRR5	0.51599747	NM_001017528	TC2200009283.hg.1
MCM3	0.49204892	NM_001270472	TC0600012038.hg.1
DBT	0.53509057	NM_001918	TC0100015061.hg.1
LRRC26	0.1923853	NM_001013653	TC0900012292.hg.1
smeysmar	0.5170932	smeysmar.aAug10-unspliced	TC1700012031.hg.1
SNTA1	0.54773855	NM_003098	TC2000008885.hg.1
FBXO34	0.537406	NM_017943	TC1400007232.hg.1
PWP2	0.461161	NM_005049	TC2100008538.hg.1
SMARCC1	0.6105502	NM_003074	TC0300010990.hg.1
ADPGK	0.48194772	NM_031284	TC1500009973.hg.1
PIK3R1	0.5444208	NM_001242466	TC0500007641.hg.1
RP5-1087E8.2	0.47500125	ENST00000438895	TC0100017546.hg.1
UBE2J1	0.44526744	NM_016021	TC0600012542.hg.1
SMAD6	0.28079492	NM_005585	TC1500007619.hg.1
DPP4	0.34977126	NM_001935	TC0200014764.hg.1
DICER1	0.36728668	NM_001195573	TC1400010068.hg.1
kawcheyby	0.4223753	kawcheyby.aAug10-unspliced	TC0800011977.hg.1
zyblu	0.3723442	zyblu.aAug10-unspliced	TC1200012275.hg.1
HIST1H2BK	0.3872197	NM_001312653	TC0600011185.hg.1
SLC25A11	0.47004586	NM_001165417	TC1700009538.hg.1
PCBP2	0.54769474	NM_001098620	TC1200012636.hg.1
bylee	0.5562395	bylee.aAug10-unspliced	TC2000007186.hg.1
CKBP1	0.56606174	OTTHUMT00000430620	TC1600010176.hg.1
DLST	0.45699555	NM_001244883	TC1400007691.hg.1
steesku	0.5435216	steesku.aAug10-unspliced	TC1800009032.hg.1
noswy	0.32253882	noswy.aAug10-unspliced	TC1500008247.hg.1
swujor	0.6279067	swujor.aAug10-unspliced	TC1900011045.hg.1
roysweebu	0.51977044	roysweebu.aAug10-unspliced	TC0500008818.hg.1
SURF6	0.55545616	ENST00000629980	TSUnmapped00000280.hg.1
GSR	0.40518555	NM_000637	TC0800010034.hg.1
gapybu	0.4904637	gapybu.aAug10-unspliced	TC0100009931.hg.1
SRSF5	0.4984582	NM_001039465	TC1400007562.hg.1
FAM111B	0.4830927	NM_001142703	TC1100007727.hg.1
TRUB2	0.5179548	NM_015679	TC0900011634.hg.1
HIST1H2AJ	0.41969615	NM_021066	TC0600011225.hg.1
SEC24B	0.46943778	NM_001042734	TC0400008376.hg.1
PHF14	0.5191417	NM_014660	TC0700006715.hg.1
SNAP23	0.60475904	NM_003825	TC1500007034.hg.1
jusky	0.38240618	jusky.aAug10-unspliced	TC1800007441.hg.1
TLE3	0.4281667	NM_001105192	TC1500009904.hg.1
CDC25C	0.46098262	NM_001287582	TC0500012166.hg.1
HIST1H2AH	0.5278977	NM_080596	TC0600014085.hg.1
ZNF625-ZNF20	0.4646631	NR_037802	TC1900011878.hg.1
CDC6	0.50786716	NM_001254	TC1700007790.hg.1
HMGN2P5	0.5414811	OTTHUMT00000417269	TC1500006692.hg.1

Gene Symbol	Log2 ratio C42B-R vs. C42B Parental	mRNA Accession	Probe Set ID
teysee	0.580512	teysee.aAug10-unspliced	TC1900008413.hg.1
SCAPER	0.61952037	NM_001145923	TC1500010097.hg.1
AURKB	0.47502816	NM_001256834	TC1700009679.hg.1
ZNF441	0.54207057	NM_152355	TC1900007045.hg.1
zeejo	0.521322	zeejo.aAug10-unspliced	TC0600007779.hg.1
TRIM37	0.5006215	NM_001005207	TC1700011273.hg.1
COG3	0.49696615	NM_031431	TC1300007070.hg.1
berrer	0.45925748	berrer.aAug10	TC1500008694.hg.1
DNAL1	0.436959	NM_001201366	TC1400007653.hg.1
PDCD6IP	0.52879274	NM_001162429	TC0300007004.hg.1
TMEM42	0.67257625	NM_144638	TC0300007225.hg.1
fernor	0.5464786	fernor.aAug10-unspliced	TC1600008535.hg.1
PLCL2	0.54020005	NM_001144382	TC0300006758.hg.1
METTL25	0.48297748	NM_032230	TC1200008318.hg.1
NMU	0.5263089	NM_001292045	TC0400010750.hg.1
TXNDC16	0.45140904	NM_001160047	TC1400009184.hg.1
YIPF1	0.5255118	NM_018982	TC0100014245.hg.1
H2AFX	0.41791686	NM_002105	TC1100012513.hg.1
RP11-290L1.4	0.3945414	ENST00000552477	TC1200008236.hg.1
TSGA10	0.44362015	NM_025244	TC0200013602.hg.1
DDX39A	0.40208858	NM_005804	TC1900009820.hg.1
TEX30	0.5548091	NM_001286775	TC1300009673.hg.1
TPR	0.50444764	NM_003292	TC0100016709.hg.1
RHPN2	0.52777475	NM_033103	TC1900010375.hg.1
RP11-326A19.5	0.20085482	ENST00000563131	TC1500010380.hg.1
STK36	0.52710193	NM_001243313	TC0200010806.hg.1
ACOX2	0.4578164	NM_003500	TC0300011309.hg.1
PAIP1	0.5039823	NM_006451	TC0500010643.hg.1
JAG2	0.43844858	NM_002226	TC1400010796.hg.1
CCBL2	0.6473662	NM_001008661	TC0100014848.hg.1
KTN1	0.49453494	NM_001079521	TC1400007244.hg.1
ZNF844	0.4110064	NM_001136501	TC1900007061.hg.1
TRIP13	0.49076706	NM_001166260	TC0500006472.hg.1
KIF18A	0.33210176	NM_031217	TC1100010418.hg.1
OXA1L	0.4004507	NM_005015	TC1400006656.hg.1
C17orf62	0.5676897	NM_001033046	TC1700012126.hg.1
LOC389831	0.45440343	NM_001242480	TCUn_GL000195v100006435.hg.1
kawklar	0.4641785	kawklar.aAug10-unspliced	TC1800006488.hg.1
gulybu	0.3822165	gulybu.aAug10-unspliced	TC0100009757.hg.1
YLPM1	0.5396027	NM_019589	TC1400010627.hg.1
SYNGAP1	0.5085994	NM_006772	TC0600014111.hg.1
GLUDP5	0.5238646	ENST00000414627	TC1000010230.hg.1
DARS2	0.538422	NM_018122	TC0100010661.hg.1
FAM21A	0.4975419	NM_001005751	TC1000007603.hg.1
swyfaby	0.3891656	swyfaby.aAug10-unspliced	TC1300006863.hg.1
swybubo	0.6070396	swybubo.aAug10-unspliced	TC0400008272.hg.1
FAAH	0.31280243	NM_001441	TC0100008158.hg.1
DNM1L	0.45855513	NM_001278463	TC1200007277.hg.1
PEG3-AS1	0.53737444	NR_023847	TC1900008972.hg.1
SRSF2	0.4972668	NM_001195427	TC1700011820.hg.1
steeleyby	0.60206133	steeleyby.aAug10	TC1000007666.hg.1
steyfar	0.6186358	steyfar.aAug10-unspliced	TC0X00009555.hg.1
ACSS1	0.49296528	NM_001252675	TC2000008722.hg.1
CYB5A	0.626406	NM_001190807	TC1800009029.hg.1

Gene Symbol	Log2 ratio C42B-R vs. C42B Parental	mRNA Accession	Probe Set ID
ATG4D	0.5405698	NM_001281504	TC1900006989.hg.1
RP11-10G15.4	0.54678494	ENST00000481228	TC0300008527.hg.1
CBS	0.54003733	NM_000071	TC2100007491.hg.1
PIGB	0.5241317	NM_004855	TC1500007303.hg.1
WNK3	0.47971934	NM_001002838	TC0X00009778.hg.1
PCBP2P1	0.6052516	OTTHUMT00000141397	TC2100008163.hg.1
KIF15	0.40996036	ENST00000625939	TSUnmapped00000127.hg.1
BDH1	0.38979703	NM_004051	TC0300013751.hg.1
snawgley	0.37326625	snawgley.aAug10-unspliced	TC1500009186.hg.1
LOC101060389	0.47515857	ENST00000620683	TC1700012242.hg.1
WDR73	0.5780801	NM_032856	TC1500010290.hg.1
ECHS1	0.5037588	NM_004092	TC1000012399.hg.1
AGO4	0.4595127	NM_017629	TC0100007784.hg.1
smeedoybu	0.4887119	smeedoybu.aAug10-unspliced	TC0200011253.hg.1
GAS2L3	0.39045474	NM_001303130	TC1200008597.hg.1
HECTD2	0.46758094	NM_001284274	TC1000008435.hg.1
SMIM4	0.68669504	NM_001124767	TC0300007485.hg.1
smaspy	0.5855923	smaspy.aAug10-unspliced	TC1600009831.hg.1
leeterbu	0.31373262	leeterbu.aAug10-unspliced	TC1800006592.hg.1
wawbleeby	0.5481077	wawbleeby.aAug10-unspliced	TC0700012626.hg.1
ZNF30	0.48478946	NM_001099437	TC1900007826.hg.1
boyboyby	0.48922414	boyboyby.aAug10-unspliced	TC1400009941.hg.1
PPP1CA	0.54464936	NM_001008709	TC1100011331.hg.1
PTER	0.61623704	NM_001001484	TC1000006880.hg.1
SRP14	0.59209675	NM_001309434	TC1500009079.hg.1
H2BFS	0.44539672	OTTHUMT00000195634	TC2100007316.hg.1
rogoy	0.6814599	rogoy.aAug10-unspliced	TC0700013278.hg.1
SLC25A6P2	0.50913954	ENST00000497974	TC0900006921.hg.1
SSX2IP	0.57008034	NM_001166293	TC0100014762.hg.1
GNMT	0.34421772	NM_018960	TC0600008062.hg.1
PABPN1	0.55949104	NM_004643	TC1400010576.hg.1
IMPDH2	0.4094581	NM_000884	TC0300011047.hg.1
TCONS_l2_00003977	0.48355672	hsa_circ_0005184	TC1000007552.hg.1
BRI3	0.44571802	NM_001159491	TC0700013405.hg.1
GLRX2	0.43001187	NM_001243399	TC0100016789.hg.1
hirimu	0.47269163	hirimu.aAug10-unspliced	TC1000008071.hg.1
ZBTB10	0.5351074	NM_001105539	TC0800008062.hg.1
ZFP30	0.6024922	NM_014898	TC1900011935.hg.1
RTKN2	0.39857036	NM_001282941	TC1000010758.hg.1
kyspabu	0.31103367	kyspabu.aAug10-unspliced	TC0600011636.hg.1
gleyjyby	0.3509167	gleyjyby.aAug10-unspliced	TC1200011372.hg.1
LYPLA1P3	0.5955728	ENST00000402650	TC0600008481.hg.1
FBXW5	0.4641971	NM_018998	TC0900012021.hg.1
SLC25A5	0.51234466	NM_001152	TC0X00008249.hg.1
TRIM49D2	0.5248753	NM_001105522	TC1100011917.hg.1
snarnerbu	0.46307427	snarnerbu.aAug10-unspliced	TC0100014779.hg.1
SENP6	0.6172269	NM_001100409	TC0600008562.hg.1
PANK2	0.553953	NM_024960	TC2000009885.hg.1
EXO1	0.42413324	NM_003686	TC0100012172.hg.1
vojaw	0.47500482	vojaw.aAug10-unspliced	TC0600011558.hg.1
CBLN2	0.6803577	NM_182511	TC1800009309.hg.1
AFF4	0.6317016	NM_014423	TC0500012032.hg.1
PKM	0.54437214	NM_001206796	TC1500009952.hg.1
MIR3975	0.40275946	NR_039771	TC1800007081.hg.1

Gene Symbol	Log2 ratio C42B-R vs. C42B Parental	mRNA Accession	Probe Set ID
gerdaw	0.6541237	gerdaw.aAug10-unspliced	TC1500008830.hg.1
PTPN20	0.5046494	NM_001042357	TC1000007533.hg.1
plervubu	0.36976948	plervubu.aAug10-unspliced	TC1700011739.hg.1
TAF1A	0.5528937	NM_001201536	TC0100017436.hg.1
SLC9A3R2	0.29307893	NM_001130012	TC1600006585.hg.1
gaspoy	0.61143446	gaspoy.aAug10-unspliced	TC1500009187.hg.1
CIT	0.5678157	NM_001206999	TC1200012143.hg.1

Table S2. Significantly deregulated genes found in docetaxel-resistant LNCaP^R cells compared to the matched parental LNCaP cell line.

Gene Symbol	Log2 ratio LNCaP-R vs. LNCaP Parental	mRNA Accession	Probe Set ID
PXN	3.56443	NM_001080855	TC1200012153.hg.1
blafleybu	3.1122556	blafleybu.aAug10-unspliced	TC1100007077.hg.1
PIK3R3	5.406014	NM_001114172	TC0100014040.hg.1
BLNK	3.5023136	NM_001114094	TC1000011515.hg.1
TM4SF1	3.545013	NM_014220	TC0300012773.hg.1
TM7SF3	2.4072413	NM_016551	TC1200010201.hg.1
DUSP16	2.95504	NM_030640	TC1200009967.hg.1
ZNF560	2.5174682	NM_152476	TC1900009584.hg.1
DUSP16	2.679847	ENST00000629698	TSUnmapped00000353.hg.1
ACRV1	2.395064	NM_001612	TC1100012712.hg.1
ATP2B4	2.9942753	NM_001001396	TC0100011267.hg.1
VCY1B	2.769458	ENST00000250823	TC0Y00006648.hg.1
DHRS7	2.935409	NM_016029	TC1400009337.hg.1
SCD5	2.4950082	NM_001037582	TC0400011180.hg.1
PCDHB13	4.2347374	NM_018933	TC0500008884.hg.1
FAM198B	5.6217136	NM_001031700	TC0400012245.hg.1
DUSP16	3.1547987	ENST00000626461	TSUnmapped00000088.hg.1
C1QTNF3	4.827522	NM_030945	TC0500013298.hg.1
TSPAN1	3.1269867	NM_005727	TC0100008145.hg.1
C8orf34	3.3590832	NM_001195639	TC0800007888.hg.1
NBEAP1	2.6210454	NR_027992	TC1500008627.hg.1
CCDC159	2.8617265	NM_001080503	TC1900011664.hg.1
SNORD116-1	3.182337	NR_003316	TC1500006573.hg.1
ZNF98	2.962111	NM_001098626	TC1900011920.hg.1
FAT1	3.6916504	NM_005245	TC0400012670.hg.1
wawvobo	2.8540244	wawvobo.aAug10-unspliced	TC0400010009.hg.1
GSTA1	2.3901138	NM_145740	TC0600012053.hg.1
RTN1	3.6280313	NM_021136	TC1400009329.hg.1
SLC44A4	3.9985197	ENST00000229729	TC0600011464.hg.1
KDM5B	2.466685	NM_006618	TC0100016963.hg.1
CYP3A5	4.202087	NM_000777	TC0700011953.hg.1
SAT1	3.286477	NM_002970	TC0X00006799.hg.1
DUSP16	2.8812482	ENST00000628303	TSUnmapped00000374.hg.1
gogerbu	3.1548991	gogerbu.aAug10-unspliced	TC0300009346.hg.1
SLFN5	3.8060744	NM_144975	TC1700007585.hg.1
TMEFF2	4.331773	NM_001305134	TC0200015266.hg.1
APLP2	3.5663965	NM_001142276	TC1100009521.hg.1
ADAM7	3.7644048	NM_003817	TC0800007043.hg.1
goyfloby	3.8766327	goyfloby.bAug10	TC0700011263.hg.1
TNFSF15	4.8133583	NM_001204344	TC0900011300.hg.1
noychorby	2.916827	noychorby.aAug10-unspliced	TC0700007194.hg.1

Gene Symbol	Log2 ratio<br>LNCaP-R vs. LNCaP Parental	mRNA Accession	Probe Set ID
NOSTRIN	4.1041965	NM_001171631	TC0200009902.hg.1
TRIM38	6.0442314	NM_006355	TC0600007257.hg.1
MPP6	2.543276	NM_001303037	TC0700006945.hg.1
CAMKK2	3.507546	NM_001270485	TC1200012199.hg.1
GPD2	3.095948	NM_000408	TC0200016548.hg.1
RAB25	3.190944	NM_020387	TC0100010149.hg.1
smoplobu	2.6784763	smoplobu.aAug10-unspliced	TC0900009968.hg.1
rekimu	4.141984	rekimu.aAug10-unspliced	TC0700011707.hg.1
RP11-507F16.1	5.618927	ENST00000529684	TC1100012808.hg.1
KLK3	4.8988104	NM_001030047	TC1900008607.hg.1
SLC25A40	3.9717932	NM_018843	TC0700011710.hg.1
ARHGEF38	3.6656058	NM_001242729	TC0400012829.hg.1
DIO1	4.1902857	NM_000792	TC0100008366.hg.1
AZGP1	2.8848197	NM_001185	TC0700011962.hg.1
PLK2	5.4971914	NM_001252226	TC0500010835.hg.1
NETO1	3.4014995	NM_001201465	TC1800009011.hg.1
BMPR1B	5.578164	NM_001203	TC0400008183.hg.1
DAZAP2	3.2398264	NM_001136264	TC1200012629.hg.1
RYR2	3.2929828	NM_001035	TC0100012113.hg.1
AAK1	3.058343	NM_014911	TC0200012961.hg.1
MDM2	3.3790278	NM_001145337	TC1200008116.hg.1
CDK19	3.7037866	NM_001300960	TC0600012839.hg.1
GDF15	3.70373	NM_004864	TC1900007384.hg.1
TP53INP1	3.9261022	NM_001135733	TC0800011150.hg.1
snervobo	4.52474	snervobo.aAug10-unspliced	TC0400008186.hg.1
LOC731631	5.7967405	LOC731631.aAug10-unspliced	TC0700011175.hg.1
PCDHB5	5.765707	NM_015669	TC0500013241.hg.1
slersweebu	19,323046	slersweebu.aAug10-unspliced	TC0400009613.hg.1
GLS2	7.000437	NM_001280796	TC1200010921.hg.1
TRPM8	4.079079	NM_024080	TC0200011147.hg.1
PALMD	4.425948	NM_017734	TC0100009183.hg.1
SH3BGRL	7.9054246	NM_003022	TC0X00007744.hg.1
slukeebu	7.0748277	slukeebu.aAug10-unspliced	TC0300013147.hg.1
GULP1	5.202367	NM_001252668	TC0200010236.hg.1
ACOX2	5.0355625	NM_003500	TC0300011309.hg.1
C1QTNF3-AMACR	8.091464	NR_037951	TC0500013296.hg.1
ANK3	4.1974845	NM_001149	TC1000010727.hg.1
PCDHB14	5.4201503	NM_018934	TC0500013247.hg.1
skargluby	4.1027513	skargluby.aAug10-unspliced	TC0600011669.hg.1
BTG2	4.8146367	NM_006763	TC0100011253.hg.1
ZDHHC15	6.922595	NM_001146256	TC0X00010107.hg.1
NLGN4X	8.998397	NM_001282145	TC0X00008989.hg.1
LCP1	6.141706	NM_002298	TC1300008840.hg.1
CDKN1A	9.6522665	NM_000389	TC0600007847.hg.1
CLDN8	5.451458	NM_199328	TC2100007881.hg.1
LOC650226	26.9515	NR_029420	TC0700011180.hg.1
AMACR	8.110192	NM_001167595	TC0500013297.hg.1
GLYATL1	6.244707	NM_001220494	TC1100013009.hg.1
nona	8.164134	nona.aAug10-unspliced	TC0700011177.hg.1
RP11-310H4.6	14.055634	ENST00000432235	TC0700011150.hg.1
ADAMTS1	8.51847	NM_006988	TC2100007821.hg.1
ROR1	13.5492325	NM_001083592	TC0100008594.hg.1
SESN3	13.418033	NM_001271594	TC1100012019.hg.1
RP11-945A11.1	6.5589886	ENST00000534068.1	TC1100007088.hg.1

Gene Symbol	Log2 ratio LNCaP-R vs. LNCaP Parental	mRNA Accession	Probe Set ID
SYT4	26.85892	NM_020783	TC1800008550.hg.1
OR51E1	58.01366	NM_152430	TC1100006663.hg.1
ABCB1	10.353241	NM_000927	TC0700011706.hg.1
FIRRE	17.908964	NR_026975	TC0X00010826.hg.1
skoyplorby	42.785618	skoyplorby.aAug10-unspliced	TC0400012717.hg.1
MAGEC2	57.19087	NM_016249	TC0X00011001.hg.1
RUNDC3B	174.82712	NM_001134405	TC0700008261.hg.1
TNFSF10	392,64548	NM_001190942	TC0300013146.hg.1
PTGFR	0.04011484	NM_000959	TC0100008812.hg.1
GNAI1	0.024177328	NM_001256414	TC0700008181.hg.1
DEPDC1	0.040950254	NM_001114120	TC0100014543.hg.1
ADAMTSL3	0.07627707	NM_001301110	TC1500008127.hg.1
BEND4	0.14824328	NM_001159547	TC0400010553.hg.1
PCDH7	0.035351582	NM_001173523	TC0400007169.hg.1
SLC22A3	0.08989562	NM_021977	TC0600010078.hg.1
EFNA5	0.101759985	NM_001962	TC0500011648.hg.1
PEG3	0.11878157	NM_001146184	TC1900011542.hg.1
TMEM45A	0.15066868	NM_018004	TC0300008148.hg.1
BLMH	0.22832285	NM_000386	TC1700010274.hg.1
HENMT1	0.17933251	NM_001102592	TC0100015171.hg.1
ANKRD18A	0.14581032	NM_147195	TC0900010056.hg.1
LRRN1	0.106709786	NM_020873	TC0300006465.hg.1
LPAL2	0.17920749	NR_028092	TC0600013780.hg.1
POT1	0.32487112	NM_001042594	TC0700012479.hg.1
ATP1B1	0.13123657	NM_001677	TC0100010543.hg.1
FAM72B	0.23223388	NM_001100910	TC0100015509.hg.1
chysloby	0.17447013	chysloby.aAug10-unspliced	TC0500011649.hg.1
NMU	0.21303496	NM_001292045	TC0400010750.hg.1
RCN3	0.24691214	NM_020650	TC1900008544.hg.1
KCNQ5	0.20526414	NM_001160130	TC0600008509.hg.1
HIST1H2BG	0.21723571	NM_003518	TC0600011136.hg.1
STXBP5L	0.2826764	NM_001308330	TC0300008524.hg.1
MAOA	0.31229696	NM_000240	TC0X00007053.hg.1
SEMA6A	0.15992631	NM_001300780	TC0500011775.hg.1
LIN7A	0.24104224	NM_004664	TC1200011385.hg.1
MAP1B	0.07084352	NM_005909	TC0500007738.hg.1
GBE1	0.30894694	NM_000158	TC0300011651.hg.1
UGT2B15	0.1385326	NM_001076	TC0400012924.hg.1
KIF20B	0.31401816	NM_001284259	TC1000008406.hg.1
MYNN	0.24139714	NM_001185118	TC0300009451.hg.1
DSEL	0.27489787	NM_032160	TC1800008952.hg.1
SH3RF1	0.2832188	NM_020870	TC0400012378.hg.1
skersworby	0.24907549	skersworby.aAug10-unspliced	TC0300008083.hg.1
RDH10	0.15124539	NM_172037	TC0800007957.hg.1
FAM72D	0.317396	NM_207418	TC0100009723.hg.1
HIST1H1B	0.21291079	NM_005322	TC0600011232.hg.1
forskarbu	0.28344628	forskarbu.aAug10-unspliced	TC0800010055.hg.1
DGKH	0.31261846	NM_001204504	TC1300006987.hg.1
CDH26	0.1793008	NM_021810	TC2000007943.hg.1
FAM72A	0.28322527	NM_001123168	TC0100017094.hg.1
zorreybo	0.27864584	zorreybo.aAug10-unspliced	TC0100008257.hg.1
MIR548T	0.24622196	NR_036093	TC0400009324.hg.1
FAM213A	0.2520724	NM_001243778	TC1000008234.hg.1
UGT2B17	0.17604344	NM_001077	TC0400012923.hg.1

**Gene Symbol**	**Log2 ratio**<br>LNCaP-R vs. LNCaP Parental	**mRNA Accession**	**Probe Set ID**
XAGE1B	0.31227544	NM_001097594	TC0X00007285.hg.1
ploroby	0.24817838	ploroby.aAug10-unspliced	TC1000006474.hg.1
CENPJ	0.38357082	NM_018451	TC1300008371.hg.1
